# *In Vitro* Lipid Digestion of Milk Formula with Different Lipid Droplets: A Study on the Gastric Digestion Emulsion Structure and Lipid Release Pattern

**DOI:** 10.1021/acs.jafc.4c05114

**Published:** 2024-10-28

**Authors:** Pu Zhao, Xue Yang, Junai Gan, Ingrid Renes, Evan Abrahamse, Nana Bartke, Wei Wei, Xingguo Wang

**Affiliations:** a State Key Laboratory of Food Science and Resources, Jiangnan University, Wuxi 214122, China; b Collaborative Innovation Center of Food Safety and Quality Control in Jiangsu Province, School of Food Science and Technology, Jiangnan University, Wuxi 214122, China; c Life Science, Danone Open Science Research Center, Shanghai 201204, China; d Danone-Jiangnan University Lipidomics & Health Innovation Center, Wuxi 214122, China; e Danone Research & Innovation, Uppsalalaan 12, Utrecht, CT 3584, The Netherlands

**Keywords:** milk formula, microstructure, *in vitro* digestion, lipolysis product, lipid droplet, digestive kinetics

## Abstract

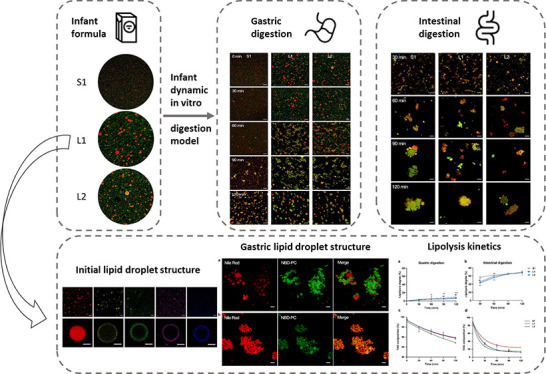

In this study, the digestive properties of milk formulas (two concept milk formulas L1 and L2 with *D*_4,3_ ∼5 μm and a control milk formula S1 with *D*_4,3_ ∼0.5 μm) were evaluated using a dynamic digestion model simulating the infant gastrointestinal tract. The results showed that L1 and L2 had a lower lipolysis degree compared to S1 during gastric digestion and no significant difference at the end of the digestion process. Triacylglycerol lipolysis products were highly related to the lipid sources of milk formulas. At the end of digestion, glycerophospholipids in milk formulas were hydrolyzed to lysophospholipids (∼60–80%), while sphingomyelins were barely hydrolyzed. Concept milk formulas showed a complete spherical structure with a mean size of 3–5 μm during gastric digestion, while the control formula had large aggregates consisting of small lipid droplets. This study reveals that the structure of lipid droplets moderates the gastric digestion emulsion structure and further influences the digestive properties of milk formulas.

## Introduction

As an important component of human milk, fat provides ∼50% of the energy needed by infants during their first months of life.^1^ It also contains essential fatty acids, fat-soluble vitamins, and cholesterol. Human milk fat exists in the form of human milk fat globules (MFGs), with a trilayer membrane known as the milk fat globule membrane.^2^ The mean size of human MFGs is 3–5 μm with a wide range of 0.2–15 μm, and the thickness of its membrane is approximately 10–20 nm.^3^ The membrane of human MFGs has been reported to have a spatial heterogeneity with the lateral segregation of sphingomyelin (SM) in liquid-ordered phase domains, which are surrounded by a liquid-disordered phase composed of glycerophospholipids.^4^

Human milk is generally regarded as the gold standard for the design of infant milk formulas. In the aspect of lipids, simulation of human breast milk in milk formulas so far was mainly focused on the fatty acid composition. In the recent decades, triacylglycerol (TAG) species have received increasing attention as they account for 98% of total lipids, whose distribution of fatty acids had an important impact on infant digestion and absorption.^5^ Very recently, studies have paid attention to the fat globule structure, in which the lipid droplet structure in milk formula showed a profound difference from human MFGs in both size distribution and interface composition. The mean particle size of lipid droplets of commercial milk formulas is typically less than 1 μm, resulting in an approximately 10-fold larger specific surface area (SSA) than human MFGs. The milk formula lipid droplet interfacial layer is composed of milk proteins and is thicker than the membrane of human MFGs. Additionally, proteins present in the aqueous phase tend to adsorb onto the interface during processing and forming an even thicker membrane.^6^ Differences in size and interfacial composition have been shown to affect lipid digestion,^7^ as this reaction takes place on the interface of the lipid droplets.

Recently, a concept infant milk formula (concept IMF) with large lipid droplets (3–5 μm) coated by cow’s milk phospholipids simulating the human MFG size, composition, and structure was developed.^8^ The concept IMF has recently shown nutritional benefits including lower fat accumulation and better metabolic profile during adulthood in mice fed with concept IMF in early life compared to control milk formula.^9^ The nutritional programming effects as displayed by an improved hepatic oxidative capacity, mitochondrial fusion, and lipid profiles persisted under a high-fat diet environment in mice.^10^ Randomized, controlled, double-blind equivalence trials indicated that concept IMF could supports adequate growth and is well tolerated in healthy, term infants,^11,12^ increased cognitive performance and BMI outcomes at school age.^13,14^ Gastrointestinal handling of concept IMF has slower gastric digestion and a lower lipid bioaccessibility rate compared to control, which may promote lipid oxidation over storage, benefiting metabolism, growth, and brain development.^15^

The nutritional benefits of concept IMF, which has a lipid droplet structure mimicking the one of human milk, might be attributable to distinct lipid digestion kinetics compared to those of conventional milk formula. Previous results showed that human milk had a lower lipolysis degree (LD) in the gastric phase, but a higher LD than commercial milk formulas at the end of the intestinal phase during *in vitro* digestion.^16^ However, additional factors, such as lipid composition and bile salt-stimulated lipase in human milk,^17^ also affect lipid digestion behavior. Differences in lipid droplets between concept IMF and conventional milk formulas, including the particle size^18^ and interface composition, have been shown previously to be important factors influencing the digestive processing.^19^ Smaller droplets generally showed a higher digestibility than large ones, with higher initial lipolysis rate and higher amount of free fatty acid (FFA) release during gastrointestinal digestion.^20^ Milk fat globule membrane (MFGM) ingredients incorporated on the lipid droplet surface of a plant-based milk formula lead to an increased LD in intestinal digestion compared to MFGM free plant-based milk formula, illustrating that MFGM components can exert their function on the lipid droplet surface and make lipolysis more similar to human milk.^17^ Milk formula with 1,3-dioleic-2-plamitate-structured lipid and MFGM ingredients with a mean particle size of 3.67 μm showed high levels of LD and FFA that were similar to human milk after intestinal digestion.^7^ However, the overall evidence of the impacts of the milk fat globule size and structure on phospholipid digestion and the consequent fate of their metabolites has not been fully revealed. Most of the studies focused on O/W emulsions; however, milk formulas containing complex macro- and micronutrient composition along with different fat globule lipid composition, droplet sizes, and structures need to be explored in details.

This study aims to compare the digestive properties of milk formulas with large phospholipid-coated lipid droplets and milk formulas with similar lipid composition but nanosize lipid droplets, evaluated using dynamic *in vitro* simulation of infant gastrointestinal lipid digestion. Comprehensive lipid analyses were applied to both TAG and phospholipid lipolysis products. Structural changes including particle size distribution, zeta-potential, and microstructure were characterized during digestion. The lipolysis data were fitted using kinetic modeling. Our results show that the microstructure of the lipid in milk formula affects infant gastric digestion, which in turn affects the intestinal lipolysis. This study provides a scientific basis for the molecular mechanisms underlying the lipid droplet structures, moderating milk lipid digestion.

## Materials and Methods

### Materials

Two concept milk formulas (L1 and L2) and one control milk formula (S1) were supplied by Danone Nutricia Early Life Nutrition (Shanghai, China). L1 and S1 contained the same lipid sources, including mixed vegetable oils, while the lipid composition of L2 was a mixture of anhydrous milk fat and vegetable oils. Proteins in milk formulas were provided with skim milk powder and whey powder. Phospholipids in milk formulas were provided by MFGM ingredients. Three milk formulas were all supplemented with the MFGM enriched ingredient extracted from bovine milk labeled as whey protein concentrates.

Rabbit gastric extract (RGE-15U) was obtained from Lipolytech (Marseille, France). Porcine bile (S31320) was purchased from Yuan Ye Bio-Technology Co., Ltd. (Shanghai, China). Pancreatic lipase (P7545), a standard mixture of 37 kinds of fatty acid methyl esters (18919-1AMP), standard triphenyl phosphate (TPP, ≥99%), 1,2-diacyl-*sn*-glycero-3-phospho-l-serine (>97%), and 9-diethylamino-5*H*-benzoalphaphenoxazine-5-one (Nile red) were obtained from Sigma-Aldrich (Shanghai, China). 1,2-Dimyristoyl-*sn*-glycero-3-phosphoethanolamine (>98%), l-alpha-phosphatidylglycerol (>99%), 1,2-dipalmitoyl-*sn*-glycero-3-phosphatidylcholine (>98%), l-alpha-phosphatidylinositol (>98%), 1-palmitoyl-2-oleoyl-*sn*-glycero-3-phosphatidic acid (>98%), sphingomyelin (>98%), and 1-oleoyl-2-hydroxy-*sn*-glycero-3-lysophosphatidylcholine (>99%) were purchased from Larodan Fine Chemicals AB (Malmö, Sweden). 1-Oleoyl-2-hydroxy-*sn*-glycero-3-lysophosphatidylethanolamine (>99%) and 1-palmitoyl-2-*sn*-glycero-3-phosphocholine (16:0–12:0 NBD-PC) were purchased from Avanti Polar Lipids (Alabaster, AL, USA). Wheat germ agglutinin Alexa fluor 488 (WGA) was obtained from Thermo Fisher Scientific (Shanghai, China). Solid-phase extraction cartridges (1 g of Si 60 silica gel, 6 mL, particle size of 55–75 μm) were obtained from ANPEL Laboratory Technologies (Shanghai, China). Methanol (MeOH), *iso*-propanol, *n*-hexane, and acetonitrile were HPLC-grade and purchased from J&K Scientific (Beijing, China). Analytical-grade anhydrous chloroform (CHCl_3_), petroleum ether, ammonium hydroxide, ethanol, diethyl ether, potassium chloride, calcium chloride, sodium chloride, and silica gel TLC plate (20 × 20 mm) were purchased from Sinopharm Chemical Reagent Co., Ltd. (Shanghai, China).

### Lipid Extraction

Milk formulas were prepared as milk emulsions by dissolving 13.5 g of formula powder in 100 mL of distilled water (40 °C), which were also used as the subsequent testing samples. Lipids present in the milk formulas were extracted using Folch’s method.^21^ Briefly, 4.5 mL of milk formula was mixed with 30 mL of CHCl_3_/MeOH (2:1, *v*/*v*). Sonication was performed for 10 min with an ice–water bath. Then, 7.5 mL of 0.9% NaCl was added. The mixtures were centrifuged at 8000 rpm for 10 min. The lower layer was transferred into a new tube, and the upper layer was extracted for a second time with the same method as described above. The final lower layer was dried under a nitrogen stream and stored at −80 °C before analysis.

### Preparation of Fatty Acid Methyl Esters

The preparation of fatty acid methyl ester (FAME) was according to our previous study.^16^ Briefly, 20 mg of milk fat was suspended in 1 mL of *n*-hexane and esterified with 0.5 mL of 0.5 mol/L KOH–CH_3_OH, and the tubes were placed in a 70 °C water bath for 10 min. A 2 mL boron trifluoride-MeOH (1:3, *v*/*v*) was added, and the tubes were incubated in a 70 °C water bath for 30 min. Then, 2 mL of *n*-hexane and 4 mL of saturated sodium chloride were added to the mixture. The upper phase was filtered by a 0.22 μm organic filter and transferred to a gas vial before analysis.

### Preparation of 2-Monoacylglycerol

Milk fat (80 mg) was mixed with 7 mL of Tris-HCl buffer (pH = 7.6), 1.75 mL of sodium cholate hydrate (0.05%), and 0.7 mL of CaCl_2_ (2.2%) in a tube. Then, 80 mg of pancreatic lipase (L3126) was added, and the tube was incubated in a water bath (37 °C) for 3 min. The hydrolysis products were separated using a TLC plate (silica gel 60, 20 cm × 20 cm), and the plate was developed with a developing solvent mixture, which is *n*-hexane/diethyl ether/acetic acid (50:50:1, *v*/*v*/*v*). The separated *sn*-2 MAG was scraped off, extracted, derivatized to *sn*-2 FAMEs, and then analyzed by GC according to our previous study.^22^

### Fatty Acid Composition Analysis

Both fatty acids and 2-monoacylglycerol were analyzed by Agilent 7820A GC equipped with a hydrogen flame ionization detector (Agilent, Santa Clara, CA, USA) and a DB-Fast FAME column (30 m × 0.25 mm × 0.25 μm, Agilent, USA), according to our previous studies.^16,23^ The carrier gas was nitrogen with a flow rate of 1.2 mL/min. The oven temperature started at 60 °C (hold for 3 min), increased to 175 °C at 5 °C/min (hold for 15 min), and then increased to 220 at 2 °C/min (hold for 10 min). The FAMEs were identified by comparison of the retention times of the sample peaks with those of FAME standards, quantified and expressed relative (wt % of total fatty acids) based on the peak area of each FAME.

### Triacylglycerol Identification and Quantification Using LC-MS

TAG species of milk formulas were identified and quantified using a UPLC system (Waters, Milford, MA, USA) coupled with quadrupole time-of-flight mass spectrometry (UPLC-Q-TOF-MS, Waters, Milford, MA, USA). The TAG molecules were separated by a UPLC BEH C18 column (2.1 mm × 50 mm × 1.9 μm, Waters, USA) at 45 °C. Mobile phase A was acetonitrile/isopropanol (1:9, *v*/*v*), and mobile phase B was 40% acetonitrile. The detection condition details and quantifications were as described in previous studies.^24^

### Triacylglycerol Lipolysis Product Analysis

Molecular species of TAG in milk formula and digesta were analyzed by a UPLC-Q-TOF-MS (Waters, Milford, MA, USA) with a BEH C18 column (2.1 mm × 150 mm × 1.7 μm, Waters, Milford, MA, USA) as previously reported.^16^ Each sample was injected twice in which TAGs, DAGs, and MAGs were detected in a positive potential mode and FFAs were detected in a negative potential mode. The detailed conditions were described in our previous study.^16^ Data were processed and analyzed by using Waters MassLynx (Waters, Milford, MA, USA).

### Phospholipid Analysis Using NMR

Phospholipid composition was analyzed according to our previous study.^25^ Sample fat (100 mg) was mixed with 0.4 mL of TPP (5 mg dissolved in 50 mL of CDCl_3_), 0.5 mL of MeOH, and 0.5 mL of EDTA-Cs^+^ solution (0.2 mol/L, pH = 7.0) in a tube. The mixture was oscillated for 1 min and then centrifuged at 4000*g* for 5 min at room temperature. Afterward, the CDCl_3_ phase (lower phase) was transferred to an NMR tube and analyzed using a Bruker Avance III-600 spectrometer (Bruker BioSpin, Billerica, MA, USA). Phospholipids in samples were identified by the relative chemical shifts related to the TPP internal standard (δ = −17.8) according to the PL standards.

### Phospholipid Lipolysis Product Analysis

The extraction of phospholipids in milk formula and digesta were according to our previous study.^26^ Briefly, 50 mg of sample lipid was dissolved in 1 mL of CHCl_3_/MeOH (4:1, *v*/*v*) and purified by SPE, which was activated with 10 mL of *n*-hexane. The nonpolar lipids were first eluted with 5 mL of *n*-hexane/ether (50:1, *v*/*v*) and 3 mL of *n*-hexane/ether (6:1, *v*/*v*). Then, the polar lipid fraction was obtained in three steps, in which 1 mL of *n*-hexane/ether (1:1, *v*/*v*) was added, followed by 6 mL of MeOH and then 3 mL of CHCl_3_/MeOH/H_2_O (3:5:2, *v*/*v*/*v*). After removal of the solvent, the polar lipid fraction was obtained and redissolved in 2 mL of CHCl_3_/MeOH (2:1, *v*/*v*). The molecular species of phospholipid in milk formula and digesta were analyzed using UPSFC-Q-TOF-MS with an Acquity UPC BEH column (3 mm × 150 mm × 1.7 μm, Waters, Milford, MA, USA) at 55 °C.^26^ The mobile phase was methanol/water (33:1, v/v) with 20 mM ammonium acetate. The flow rate was 1.1 mL/min, with the solvent from 5 to 48% over 5.5 min, followed by maintenance at 48% for 4.5 min, and 2 μL samples were injected for analysis. The mass spectrometer parameters were set as follows: desolvation gas flow 50 L/h, cone gas flow 700 L/h, ion source temperature 100 °C, desolvation temperature 400 °C, cone voltage 30 eV, low collision energy 6 eV, high collision energy range 20–45 eV, and scan range 100–1600 *m*/*z*. The data were analyzed by comparing the obtained *m*/*z* values and fragment data with calculated exact masses using the software Progenesis QI (Nonlinear Dynamics, Waters, MA, USA) equipped with a local database and LipidBlast database.

### Dynamic *In Vitro* Simulation of Infant Gastrointestinal Lipolysis

Gastrointestinal digestions of milk formulas were performed using an *in vitro* dynamic system simulation of term infant as described in our previous publication.^27^

#### Digestion Conditions

Milk formula emulsions (100 mL) were used in this study. The volumes of simulated gastric fluid (SGF) with RGE, simulated intestinal fluid (SIF) with pancreatin, and bile salts were adjusted by adjusting the rate of pumps to maintain the food-to-digestive juice ratio according to our previous published study.^27^ The digestion procedure was performed at 37 °C by using a circulating water bath. Digestion duration was 120 min, and 12 mL of digesta samples was obtained at 0, 30, 60, 90, and 120 min from both gastric and intestinal phases. Structural characterization of the digesta sample (2 mL) was performed immediately. For lipid composition analysis, HCl (10 mol/L, 0.02 mL) was added in each 5 mL of digesta to stop lipolysis, and lipids were extracted and stored at −20 °C until analysis.

#### Lipolysis Degree

The lipid classes including TAGs, diacylglycerols (DAGs), monoacylglycerols (MAGs), and FFAs were analyzed according to our previous studies.^16^ The degree of lipolysis (LD) is calculated by the following equation:
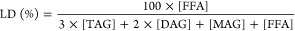
where LD (%) represents the molar percentage of FFA in total acyl chains and residual glycerides at a given time. [TAG], [DAG], [MAG], and [FFA] represent the mole fractions of the corresponding components during lipolysis. Mass fractions were converted to mole fractions by using their average molecular weights.

#### Single-Response Kinetic Modeling

Lipolysis kinetics during both the gastric and intestinal phase were evaluated via an empirical, fractional conversion model to compare their digestion behaviors.^28^ The kinetic parameters were estimated with the following equation:
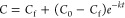
where *C* (%) represents the response at time *t* (min) during the digestion phase, i.e., the actual detected content of TAG (%) at each time point during the gastrointestinal digestion; *C*_f_ (%) represents the estimated plateau value, i.e., the evaluated content of TAG (%) at G120 and I120; *C*_0_ (%) is the initial value (*t* = 0), i.e., the evaluated initial content of TAG at G0; and *k* (min^–1^) is the estimated reaction rate constant. The estimated kinetic parameters, *C*_f_ (%) and *k* (min^–1^), were compared by calculating confidence intervals (95%). Additionally, the lipolysis rates *r* at different time points were also calculated according to the slope at each time point.

### Size Distribution and Zeta-Potential

The structures of milk formulas before and after digestion were observed according to our previous studies.^29^ The particle size and distribution of the milk and digesta samples were analyzed using a Microtrac S3500 laser light scattering (Microtrac Instruments, PA, USA). The refractive indices used for the samples and water were 1.59 and 1.33, respectively. Standard parameters of particle size, including the average volume particle size (*D*_4,3_), the average area particle size (*D*_3,2_), SSA, and distribution were calculated by the Microtrac software (Microtrac, PA, USA).

The zeta-potential values of milk and digesta samples were measured using a Malvern Zetasizer Nano ZS instrument (Malvern, UK) at room temperature (25 ± 2 °C). Samples were analyzed after being diluted 250 times, and each sample was analyzed in triplicate.

### Confocal Laser Scanning Microscope

The CLSM images of milk and digesta samples were obtained using a Zeiss LSM880 confocal laser scanning microscope (Zeiss, Oberkochen, Germany) with a 40× magnification objective lens according to our previous study.^30^ Samples were single- or dual-stained with fluorescent dyes, including Nile red (1 mg/mL ethanol) for neutral lipids and excited with the 514 nm Ar^+^ laser line and Rh-DOPE (1 mg/mL in chloroform) for polar lipids with excitation by the 543 nm He–Ne laser line; NBD-PC (1 mg/mL in chloroform) for phosphatidylcholine, NBD-SM (1 mg/mL in chloroform) for sphingomyelin, and WGA (1 mg/mL in PBS, pH = 7.0) for glycosylated molecules (mainly glycoproteins and glycolipids), which were excited with the 488 nm He–Ne laser line. Staining was carried out for 20 min in the dark, and 5 μL of sample was used on a microscope slide to observe. The CLSM images were analyzed using the ZEN lite black software (Zeiss, Oberkochen, Germany).

### Transmission Electron Microscope

The microstructures of milk formulas were also observed using an H-7650 transmission electron microscope (TEM, Hitachi High Technologies, Tokyo, Japan) according to our previous study.^29^ Milk formula emulsions were evenly mixed with 3% agarose tubes (1:1, *v*/*v*) and then stored at 4 °C overnight. Approximately 1 mm^2^ block was cut into 2.5% glutaraldehyde in 0.2 mol/L phosphate buffer (pH = 7.0). Samples were fixed with 1% osmic acid solution, dehydrated in ethanol and acetone, and then embedded in resin. The resin blocks were cut into 70–90 nm slices by an ultramicrotome (Leica EM UC7, Wetzlar, Germany), then stained with saturated uranyl acetate in 50% ethanol and lead citrate solution.

### Statistical Analysis

The TAG data were analyzed using MassLynx v4.1 software (Waters, Milford, MA, USA). Molecular species profiles of phospholipids were identified with the software Progenesis QI equipped with the Progenesis MetScope self-built local database and LipiBast database. All measurements were conducted in triplicate. Results are expressed as the mean ± standard deviation. All statistical analyses were performed using IBM SPSS statistics 25 (IBM Corp., Armonk, NY, USA). The statistical analysis of data was performed with one-way ANOVA followed by Duncan’s Multiple Range test to identify significant differences among groups (*P* < 0.05).

## Results and Discussion

### Lipid Droplet Size and Microstructure of Milk Formulas

The compositions and structural characteristics of the milk formulas investigated in this study are shown in Table 1. The total fat contents of these milk formulas were 3.41, 3.44, and 2.78 g/100 mL, respectively. There are both microstructure differences (S1 vs L1/L2) and lipid composition differences (L1 vs L2).

**Table 1:** Lipid Ingredients and Structural Characteristics of Milk Formulas^a^

formula	lipid sources	fat content (g/100 mL)	*D*_4,3_ (μm)	*D*_3,2_ (μm)	SSA (m^2^/g fat)	Zeta-potential (mV)
S1	mixed vegetable oils, DHA, ARA, MFGM ingredient	3.41 ± 0.18	0.52 ± 0.20^b^	0.36 ± 0.01^c^	16.50 ± 0.25^a^	–22.20 ± 1.05^b^
L1	mixed vegetable oils, DHA, ARA, MFGM ingredient	3.44 ± 0.21	5.20 ± 0.04^a^	3.16 ± 0.13^b^	1.90 ± 0.08^b^	–13.43 ± 1.29^a^
L2	mixed vegetable oils, anhydrous milk fat, DHA, MFGM ingredient	2.78 ± 0.13	5.18 ± 0.08^a^	3.45 ± 0.04^a^	1.74 ± 0.02^b^	–13.40 ± 0.10^a^

a The values are represented as mean ± SD. Different letters (a–c) in the row indicate significant (*P* < 0.05) differences among samples. SSA, specific surface area.

The *D*_4,3_, *D*_3,2_, SSA, and zeta-potential of milk formulas are shown in Table 1. The *D*_4,3_ values of L1 and L2 were both ∼5.20 μm, which was significantly larger than that of S1 (∼0.5 μm, *P* < 0.05) and similar to that of human MFG (4.50–5.32 μm).^25,29^ The particle size of S1 was similar to that of commercial formulas, which range from 0.25 to 0.70 μm.^6^ Additionally, there was an 8.68–9.48-fold difference in SSA between S1 and L1/L2 (*P* < 0.05). As shown in Table 1, the zeta-potential of L1 was similar to L2 (−13.43 and −13.40 mV, respectively), both of which were significantly higher than that of S1 (−22.20 mV, *P* < 0.05). Clear differences in lipid droplet particle size, size distribution, and zeta-potential were thus observed between both concept formulas (L1 and L2) and the control formula (S1).

**Figure 1:**
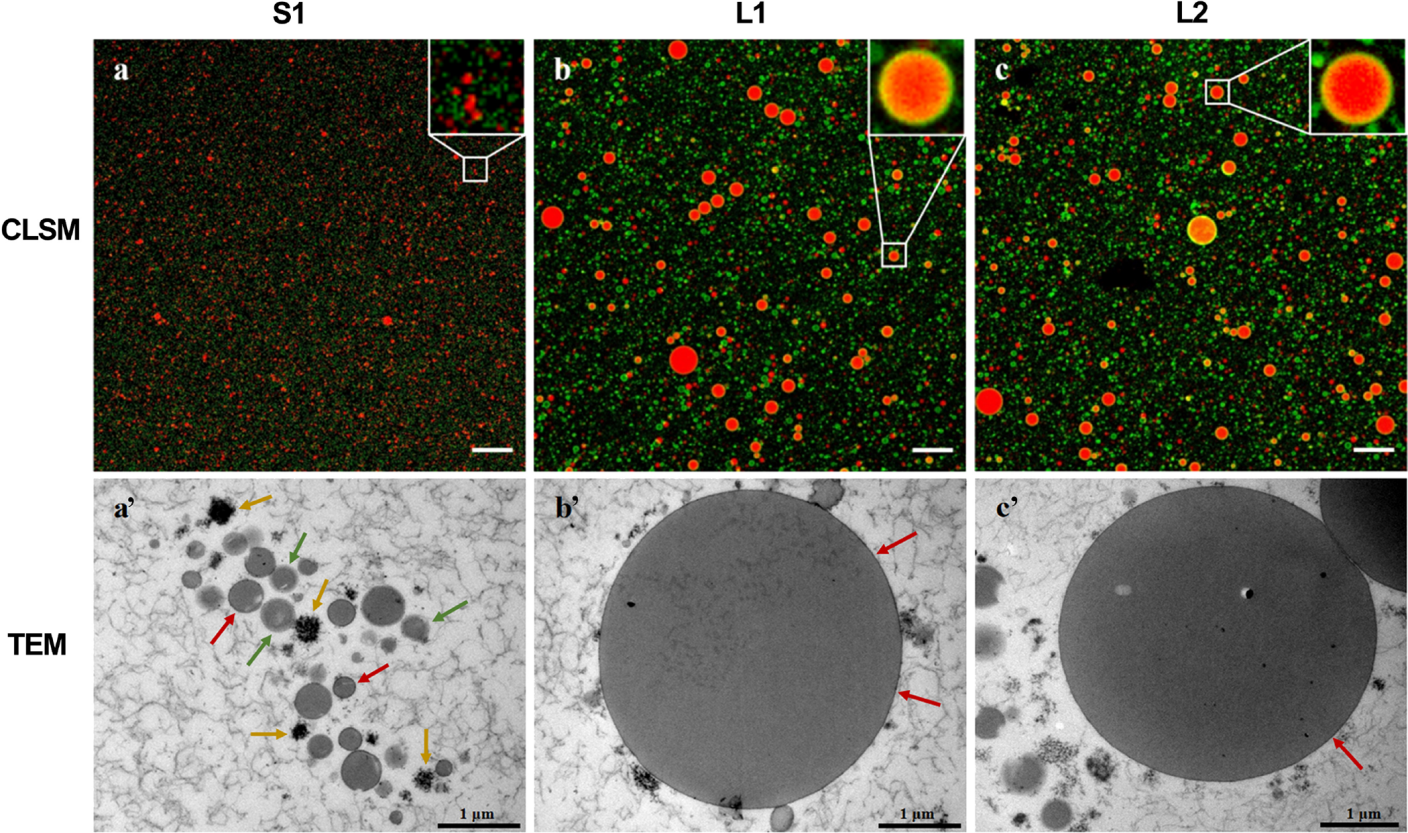
Comparison of the lipid droplet microstructure in control milk formula S1 and concept milk formulas L1 and L2. (a–c) CLSM images of lipid droplets double-stained with Nile Red and NBD-PC fluorescent probes in (a) S1, (b) L1, and (c) L2. All scale bars, 20 μm. (a’–c’) TEM images of lipid droplets in (a’) S1, (b’) L1, and (c’) L2. All scale bars, 1 μm.

The microstructure of the lipid droplets in the milk formulas in this study was investigated by using CLSM, as shown in Figure 1a–c. A clear spherical structure of lipid droplets was present in L1 and L2, whose size distributions were similar to that of human milk as previously reported.^30^ While the lipid droplets in S1 were much smaller and not completely covered by a phospholipid membrane (NBD-PC stained, green). The lipid droplet microstructures of all milk formulas were also investigated by TEM (Figure 1a’–c’). In both L1 (b) and L2 (c), a membrane mainly containing phospholipids (approximately 10 nm, red arrowheads) covering the lipid droplets was clearly observed, indicating that the MFGM ingredient formed a relatively dense membrane on the surface of the lipid droplets. While on the TEM images of S1, the composition of the oil–water interface of the lipid droplets was inhomogeneous, including droplets covered by dense MFGM ingredients (red arrowheads) and droplets covered by proteins (green arrowheads), making the membrane much thicker; additionally, some protein aggregates were observed (yellow arrowheads). Similar protein aggregates were also found in the previous TEM observation of commercial milk formulas.^6^ The inhomogeneous interface composition of S1 may be due to its larger SSA (∼17 m^2^/g fat), which poses a too large area to be covered by phospholipids alone, and thus, milk proteins become emulsifiers as well, as reported previously with commercial milk formulas where gaps were filled by proteins.^29^

**Figure 2:**
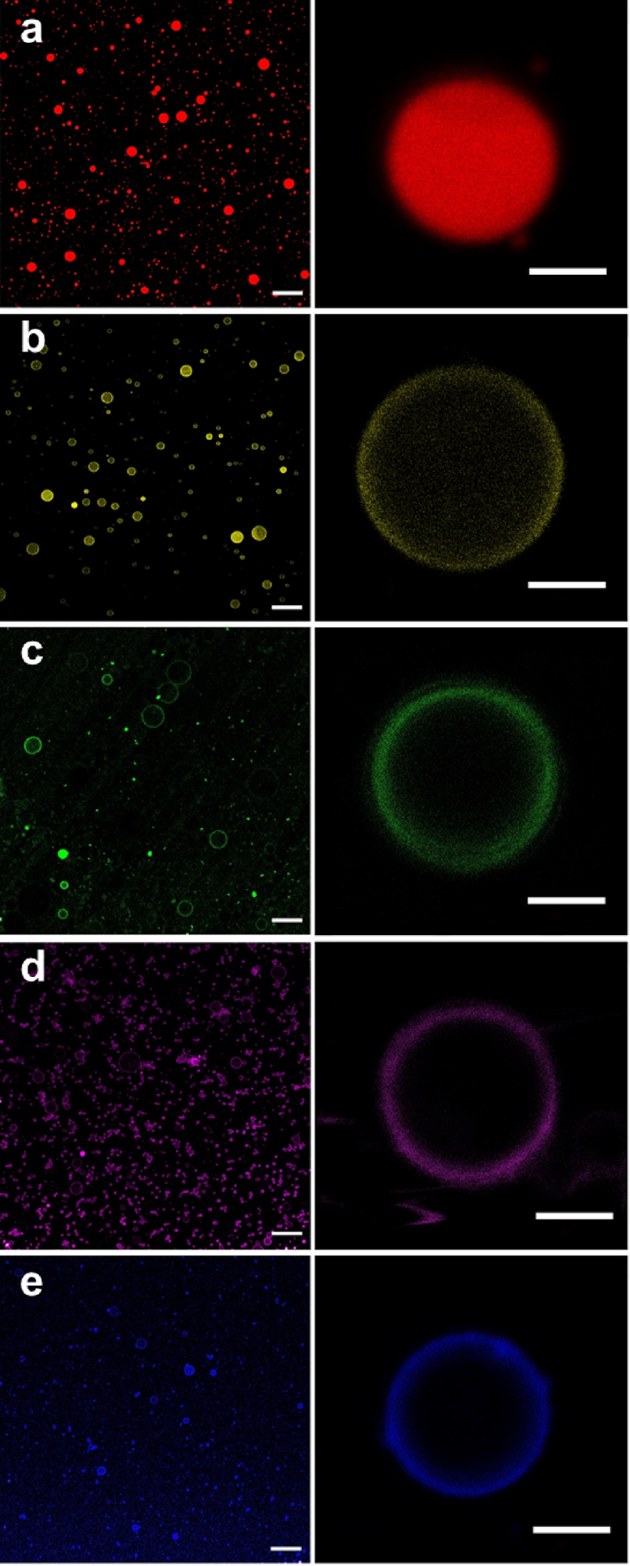
Microstructure of representative lipid droplets in concept milk formula (L1 as a representative) observed by CLSM with different fluorescent probes. (a) Phenoxazine dye Nile Red used for labeling of the neutral lipids (mainly triacylglycerols). (b) Exogenous phospholipid with Rh-DOPE labeled the polar lipids in the membrane. (c) 16:0–12:0 NBD-PC specifically labels the phosphatidylcholine in the membrane. (d) 12:0-NBD-SM specifically binds to the SM in the membrane. (e) WGA used as labeling the glycosylated molecules (glycoproteins and glycolipids). Left panel, scale bars, 20 μm; right panel, scale bars, 5 μm.

Since the overall microstructure and CLSM images are similar for L1 and L2, as shown in Figure 1b,c, L1 was chosen as a representative of the concept formulas for further observation by CLSM stained with different fluorescent probes. Five fluorescent molecules including Nile Red, Rh-DOPE, NBD-PC, NBD-SM, and WGA were used to label the neutral lipids (mainly TAGs), polar lipids, PC, SM, and glycoproteins/glycolipids, respectively. As shown in Figure 2, the microstructure of the lipid droplets in the concept milk formula consisted of a core of neutral lipids (Figure 2a, mainly TAGs) that was coated with polar lipids (Figure 2b–d) and glycoproteins/glycolipids (Figure 2e) present in the membrane. Polar lipids stained by Rh-DOPE, PC stained by NBD-PC, SM stained by NBD-SM, and glycoproteins and glycolipids stained by WGA could be observed as a clear circular structure covering the core of neutral lipids. The microstructure observed in the milk formula lipid droplets was thus very similar to the microstructure of human MFGs in both the fat globule size and interface structure.^31^

### Lipid Composition of Milk Formulas

In this study, lipid compositions, including fatty acids, *sn*-2 fatty acids, triacylglycerols, and phospholipids, were systematically analyzed in the three milk formulas.

#### Fatty Acid and sn-2 Fatty Acid Profile

In all three milk formulas, the most abundant fatty acids were saturated fatty acids (SFAs), accounting for 42–43 wt % of total FAs, followed by monounsaturated fatty acids (MUFAs), which amounted to 38–40 wt %, as shown in Table S1. Milk formulas S1 and L1 have the same lipid sources and therefore, not surprisingly, show similar fatty acid composition. L2 had a slightly higher but not significantly different content of SFA than S1 and L1, which might be due to the medium-chain fatty acids deriving from the anhydrous milk fat source.^32^ The content of polyunsaturated fatty acids (PUFAs) in milk formulas was 18–19 wt %, with L2 having slightly higher but not significantly different content than others. Moreover, the ratio of LA to ALA was 8.24–8.63 in these milk formulas, which was in the same range as human milk,^33^ and also met the general regulations (for example, the Codex Alimentarius Commission and the National Health Commission of the People’s Republic of China) requirement of 5–15.^24^

The *sn*-2 fatty acid profiles of the three milk formulas are shown in Table S2. The most abundant fatty acids at the *sn*-2 position of the glycerol backbone in all three milk formulas are MUFAs, accounting for 40–51 wt % of total fatty acids, followed by SFAs, which are 25–38 wt %. Both S1 and L1 had higher *sn*-2 MUFA and PUFA contents than L2, which may be due to the presence of the anhydrous milk fat ingredient in L2. Additionally, both S1 and L1 had considerably lower levels of *sn*-2 SFA than L2, which might be due to *sn*-2 palmitate supplementation as a consequence of the anhydrous milk fat ingredient in L2. Therefore, the SFA level at the *sn*-2 position of the glycerol backbone in this formula is closer to that of human milk fat.^34^

#### Triacylglycerols Composition

In this study, using LC-MS, a total of 156 different TAG species were detected of which 28 TAG molecular species accounted for more than 1 wt % of total TAGs, as shown in Table S3. Formula S1 showed a TAG composition similar to L1, in which 18:1/18:1/18:1 was the most abundant TAG (>15 wt %), followed by 18:1/16:0/16:0 (∼12.7 wt %) and 18:1/18:1/16:0 (12.6 wt %). Moreover, L2 had obvious lower content of 18:1/18:1/16:0 (∼5 wt %) and 18:1/16:0/16:0 (∼3 wt %) than S1 and L1, but had some unique species of TAGs, such as 18:1/14:0/4:0, 18:0/16:0/4:0, or 16:0/16:0/6:0, which are most likely deriving from the anhydrous milk fat source that typically has a high short/medium-chain fatty acid content (not shown). TAGs of 18:1/16:0/18:2 and 18:1/16:0/18:1 were reported as the first or second abundant TAGs in human milk, with contents of more than 10%.^22^ However, in the investigated milk formulas, these TAGs accounted for less than 1 wt %. Contrarily, 18:1/18:1/18:1 accounted for only ∼3 wt % in human milk,^35^ which is the main TAG in the milk formulas of this study. The results of TAGs in these milk formulas were consistent with *sn*-2 fatty acid composition described above.

#### Phospholipids Composition

The total phospholipid contents of S1, L1, and L2 were similar (approximately 17–18 mg/g fat). All formulas have higher phospholipids than what has been reported previously for human milk (∼7 mg/g fat).^29^ However, other studies also reported a dynamic total phospholipid content in human milk in time, peaking at ∼25 mg/g fat.^36^ This might be due to a physicochemical biological result of changing at fat globule size during lactation and consequently resulting in higher surface area of the fat globules, which would then require higher amounts of phospholipids in the MFGM to fully cover the fat globules. In these three milk formulas, phosphatidylethanolamine (PE, ∼29%) and phosphatidylcholine (PC, ∼28%) were the most abundant polar lipids, followed by SM (∼25%), phosphatidylserine (PS, ∼10%), and phosphatidylinositol (PI, ∼7%). These results are consistent with the order of PC/PE > SM > PI > PS in cow’s milk, but slightly different from human milk where SM is the most abundant type.^36,37^

### Lipolysis Degree and Digestion Kinetics of Milk Formulas

**Figure 3:**
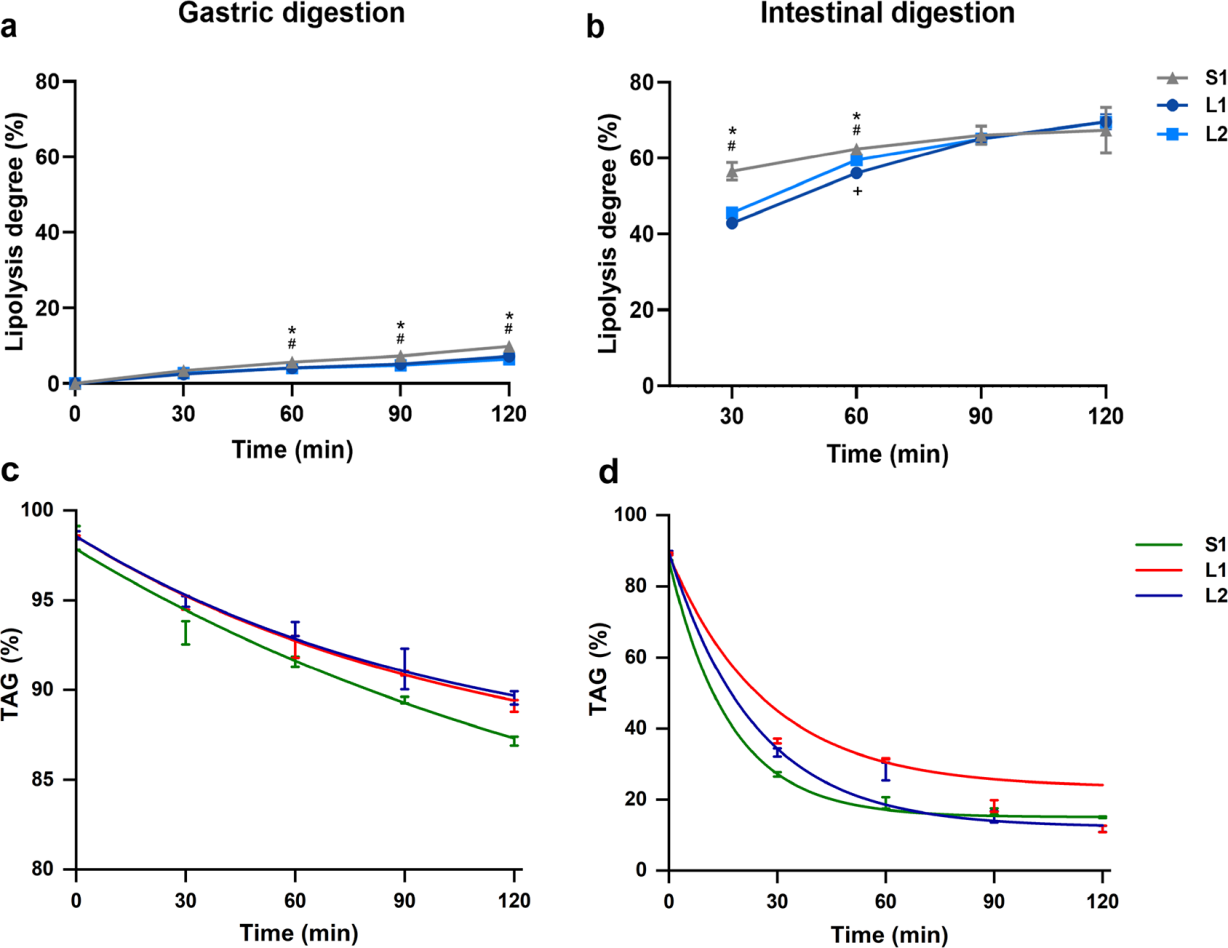
Digestion profiles of control milk formula S1 and concept milk formulas L1 and L2. Lipolysis degree (%) of milk formulas during (a) *in vitro* gastric and (b) intestinal digestion. Triacylglycerol lipolysis (% of total triacylglycerols) fitting fractional conversion model of milk formulas during (c) *in vitro* gastric and (d) intestinal digestion. The statistical analyses for each experiment are shown in the figures. *, ^#^, and ^+^ represented significant difference between S1 and L1, S1 and L2, and L1 and L2, respectively (*P* < 0.05).

The LD of milk formulas during *in vitro* gastrointestinal digestion is shown in Figure 3a,b. Formula S1 showed a significantly higher LD (*P* < 0.05) than L1 and L2 from 60 min onward (G60) during the gastric phase, while no significant differences were found between L1 and L2. At G120, the LDs of L1 and L2 were ∼7 and ∼6%, which were significantly lower than that of S1 (∼10%, *P* < 0.05, respectively). S1 with its smaller particle size and larger SSA likely provides more binding sites for gastric lipase,^38^ which explains the results described above.

The LDs of these milk formulas increased sharply once chyme was delivered to the intestinal phase, which can be attributed to the activity of pancreatic lipolytic enzymes. At the first time point of the intestinal phase (30 min, I30), the LD of S1 (∼57%) was significantly higher (*P* < 0.05) than that of L1 and L2. L2 had a significantly higher LD than L1 at I60, which might be due to the higher amounts of short/medium-chain fatty acids from anhydrous milk fat present in L2 that are more hydrophilic and might be more easily removed from the lipid droplet interface. At the end of the intestinal digestion (I120), the LDs of S1, L1, and L2 reached ∼67, ∼67, and ∼70%, respectively, showing no significant difference (*P* > 0.05).

The TAG (%) decrease during *in vitro* gastric and intestinal digestion fitted with the fractional conversion model is shown in Figure 3c and Figure 3d, respectively. TAGs are the main substrates for lipases during this digestion reaction, and their decrease rate would be reflected in the reaction rate (*k*).^18^ Additionally, the initial TAG content value (*C*_0_), the estimated plateau TAG value (*C*_f_), and the rate at different time points (*r*) were also calculated to get insights in the entire digestion process, these parameters are shown in Table 2. During the gastric digestion, S1 and L1 showed higher *k* than L2 (0.008 min^–1^, *P* < 0.05); moreover, the absolute value of *r*_*0*_ in S1 (0.175, *t* = 0) was significantly higher than that in L1 and L2, indicating a faster initial digestion rate. Both *C*_0_ and *C*_f_ were not significantly different (*P* > 0.05) between the three milk formulas, and the *r* of S1 did not consistently remain the highest at all time points (data not shown). The *r* of S1 was similar to the *r* values of L1 and L2 at G60, then higher onward, while slower from I60 onward than that of L1 and L2. This illustrates that the differences in particle size (S1 < L1/L2) influenced the reaction rate at the beginning, and that from 60 min onward product inhibition occurred in this phase, likely because there were no bile salts present that could help remove the lipolysis products from the interface.^39^ Once the chyme was delivered to the intestinal phase, again significantly different fitting parameters between milks were observed. When the digestion products were removed from the interface by the action of bile, the equilibrium of the reaction shifted toward continued lipolysis, indicating the activity of pancreatic lipolytic enzymes. Although the LD of S1 was similar to that of L1/L2 at the end of the intestinal phase, the *k* of S1 (0.061 min^–1^) was evidently higher than that of L1 and L2. This could be attributed to the catch-up phenomenon that occurred after I60, in which the absolute value of *r* in L1 and L2 (0.301–0.304) from I60 was higher than that of S1 (0.173), while it was much higher at I0 (2.002) and I30 (1.134) in S1. The *C*_f_ of S1 was higher than that of L1/L2 (*P* < 0.05), which was consistent with the LD described above (Figure 3).

**Table 2:** Fractional Conversion Model Parameter Estimates of TAG Concentration (%) in Milk Formulas during *In Vitro* Gastric and Intestinal Digestion

samples	*C*_0_ (%)	*C*_f_ (%)	*k* (min^–1^)	*r*_0_^a^	*R*^2^
*Gastric digestion*					
S1	98.20 ± 0.70	84.12 ± 0.67	0.012 ± 0.001^a^	–0.175 ± 0.001^a^	0.96735
L1	98.49 ± 0.13	85.66 ± 0.98	0.011 ± 0.001^a^^a^	–0.123 ± 0.012^b^	0.99867
L2	98.46 ± 0.37	84.15 ± 1.54	0.008 ± 0.001^b^	–0.123 ± 0.006^b^	0.98619
*Intestinal digestion*					
S1	87.11 ± 0.26^b^	16.12 ± 0.71^a^	0.061 ± 0.001^a^	–2.002 ± 0.024^a^	0.99984
L1	88.29 ± 0.38^a^	14.25 ± 0.28^b^	0.034 ± 0.001^c^	–1.755 ± 0.013^b^	0.96955
L2	89.03 ± 0.48^a^	13.98 ± 1.16^b^	0.040 ± 0.002^b^	–1.877 ± 0.050^b^	0.99658

a Different letters (a–c) in the line indicate significant (*P* < 0.05) differences of *r*_0_ among samples. *C*_0_ is the initial estimated concentration. *C*_f_ is the final estimated concentration. *k* is the estimated reaction rate constant. *r*_0_ is the initial rate being the tangent at *t* = 0, in which 120 min in the gastric phase was used as the initial time (0 min) of intestinal digestion. *R*^2^ is the coefficient of determination of the fractional conversion model.

### Lipolysis Product Release Pattern of Milk Formulas during *In Vitro* Digestion

Lipolysis products of milk formulas, comprising TAGs, DAGs, MAGs, and FFAs during *in vitro* digestion, were quantified by UPLC-MS/MS. The identification and characterization of these lipolysis product molecules will give insights into their lipolysis release pattern during digestion.

#### Triacylglycerol Lipolysis Products

**Figure 4:**
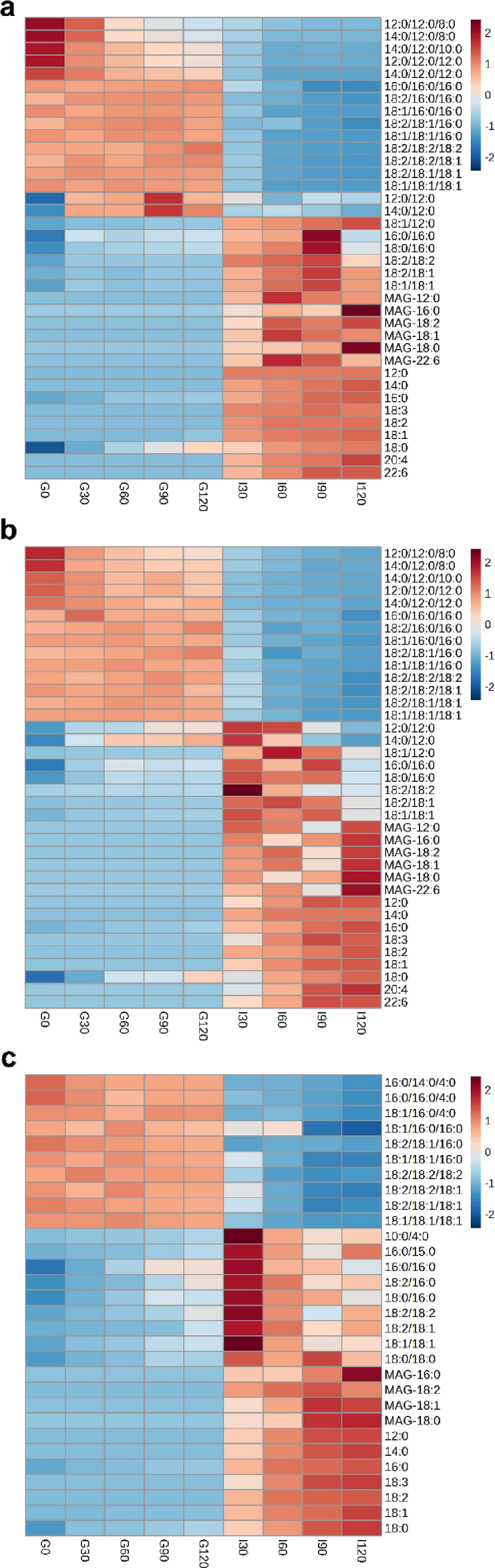
Triacylglycerols lipolysis products of control milk formula and concept milk formulas analyzed using LC-MS. Heatmap of main released free fatty acids, monoacylglycerols, diacylglycerols, and undigested triacylglycerols in S1 (a), L1 (b), and L2 (c) during *in vitro* gastrointestinal digestion.

Different milk TAGs compositions might be digested into different lipolysis product species, which could potentially affect their absorption and metabolic pathways.^40^ The species of milk TAG and their lipolysis products, including DAGs, MAGs, and FFAs released during *in vitro* gastrointestinal digestion, are shown in Tables S4–S6. A total of 105, 108, and 173 different species of digestion products were detected in S1, L1, and L2, respectively. The most abundant lipolysis product molecular species of the three milk formulas, which are 37 (S1), 37 (L1), and 30 (L2) at different time points, are displayed as heatmaps in Figure 4. Overall, the types of lipolysis products in S1 and L1 were similar, while L2 had a higher number of unique types, probably due to differences in the lipid sources. Nevertheless, the results showed similarly abundant amounts of MAGs, FFAs, and long-chain unsaturated fatty acid TAGs in the milks. This could be predicted according to the milk TAGs, as the abundant milk TAGs were similar and the specificity of lipolytic enzymes is constant during digestion.^16,41^

For milk formulas with similar lipid compositions but different lipid droplet structures (S1 vs L1), approximately 11–13 and 86–89% of TAGs were hydrolyzed at G120 and I120, respectively. DAGs (mainly 18:2/18:1, 18:1/18:1, 18:1/12:0, and 18:0/16:0) exhibited an upward trend early during digestion, followed by a downward trend later during digestion, indicating their release and subsequent digestion in the intestinal phase. MAGs (mainly MAG-18:1, 18:0, and 16:0) and FFAs increased significantly once the chyme was delivered into the intestinal phase. At the end of the entire digestion, FFA 12:0, 18:1, 18:2, 18:3, and 20:4 were higher, while 14:0, 16:0, 18:0, and 22:6 were lower in L1 than in S1 (Tables S4–S6). The elucidation of the reasons for this requires further study. Medium-chain (C_6_–C_12_) unsaturated fatty acids are reported to be easily absorbed from the gastrointestinal tract, while FFA 16:0 has been reported to be less easily absorbed; due to its tendency for soap formation with calcium, it could be excreted from the body via the feces, resulting in the loss of calcium and energy. This suggests that in the body, FFAs from L1 could be easier absorbed than from S1. Furthermore, for milk formulas with similar lipid droplet structures but different lipid compositions (L1 vs L2), the total contents of TAGs, DAGs, MAGs, and FFAs were similar during the whole *in vitro* digestion. However, the released species of DAGs (mainly 18:0/18:0, 18:0/16:0, and 18:2/18:1), MAGs (mainly MAG-18:1, 16:0, 18:0, and 18:2), and FFAs (mainly 18:1, 16:0, 18:2, and 18:0) in L2 were different, indicating that the types of digestion products were highly correlated with their lipid sources in the milk formulas, in which less 18:1/16:0/16:0, 18:1/18:1/16:0, but more 16:0/16:0/4:0 and 18:2/18:2/18:1 were found in L2.

#### Phospholipid Lipolysis Products

**Figure 5:**
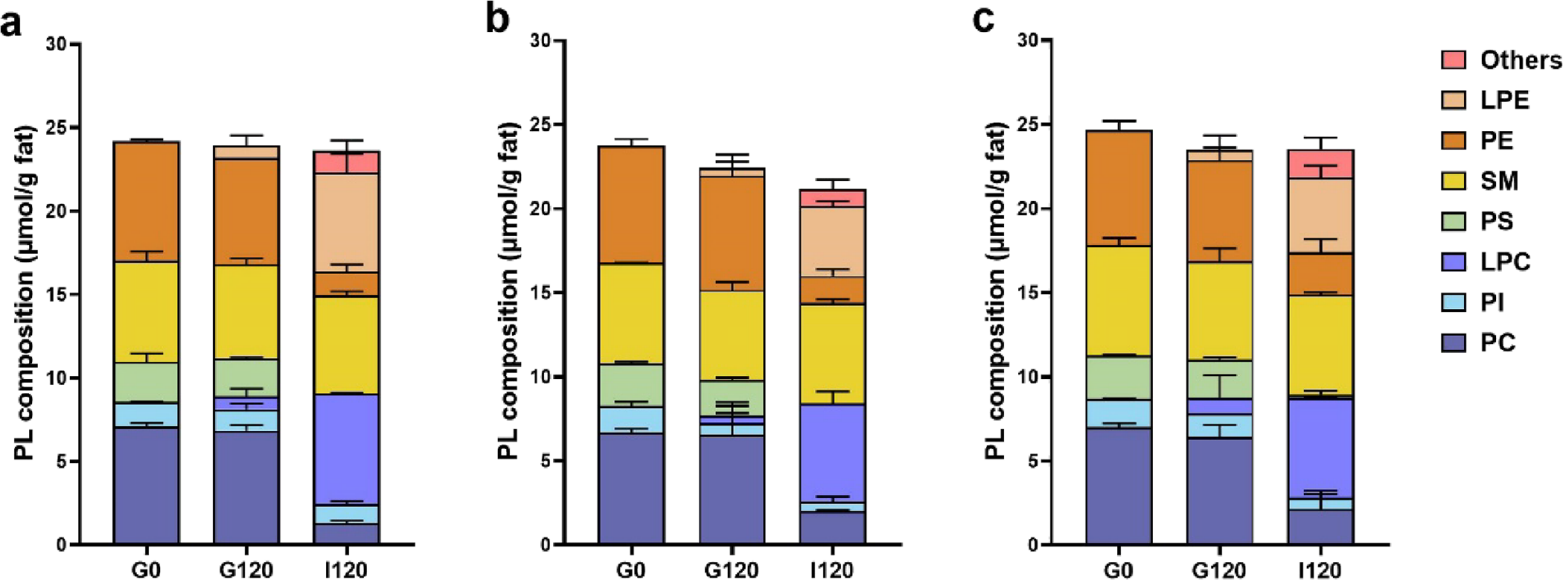
Polar lipid lipolysis product concentration (μmol/g fat) of control milk formula S1 (a), concept milk formulas L1 (b), and L2 (c) at G0, G120, and I120 during *in vitro* gastrointestinal digestion analyzed using LC-MS.

Phospholipid intake is essential for human beings because it provides choline, ethanolamine, and polyunsaturated fatty acids. Some of these are used to resynthesize phospholipids, which are crucial for physical development and health, and involved in a number of cellular functions.^42^ The phospholipid concentrations of the different milk formulas before digestion and at 120 min of gastric and intestinal phase are shown in Figure 5 and Table S7. The PC and PE contents of the different milk formulas ranged from 6.70 to 7.07 μmol/g fat and 6.82 to 7.15 μmol/g fat, respectively. At G120, small amounts of lysophosphatidylcholine (LPC) and lysophosphatidylethanolamine (LPE) were detected. At the end of the entire digestion, large amounts of LPC (∼6 μmol/g fat) and LPE (4–6 μmol/g fat) were detected, indicating that 63–82% of PC and PE were hydrolyzed. Studies have reported that the expression of pancreatic phospholipase A2 (PLA2) and mucosal phospholipase B (PLB) might be low in newborns, while pancreatic lipase-associated protein 2 (PLRP2), which is active on both PC and PE, might play a more critical role in the hydrolysis of glycerol-phospholipids in newborns.^42^

Studies also reported that porcine pancreatic lipase showed pancreatic phospholipase A1 (PLA1) activity, although it was not present in human pancreatic lipase.^43^ The porcine pancreatic lipase used in our study is secreted by the porcine pancreas and present in porcine pancreatin; thus, it may contain a mixture of enzymes besides TAG lipase, for example, PLA2. Similar to the lipolysis of neutral lipids, the hydrolysis of phospholipids is also affected by the lipid droplet structure. S1 showed more LPC and LPE than L1 and L2 at I120, which may because more phospholipids including those in aqueous phase could attach to enzymes in S1 with smaller lipid droplet size and thus larger SSA. Additionally, the PS decreased dramatically (*P* < 0.05), and PI content slightly decreased as well (*P* > 0.05). Some of the other phospholipid species found in the intestinal phase might be hydrolysis products of PS and PI. There was no significant difference found in SM before or after digestion, which could be due to the absence of SM hydrolyzing capacity in RGE or pancreatic lipase.^42^

More than 81 different species of phospholipids were detected in the milk formulas during the entire digestion (Table S7). The phospholipid profiles of all of the milk formulas were similar due to the addition of the same MFGM ingredient. The most abundant PC molecular species are PC (18:1/16:0), PC (18:1/18:1), and PC (16:0/16:0), which were hydrolyzed to LPC including PC (18:1/0:0) and PC (18:0/0:0). PE (18:2/18:0) and PE (18:1/16:0) were hydrolyzed to LPE such as PE (18:1/0:0), PE (18:0/0:0), and PE (16:0/0:0). PI (18:1/18:0) and PS (20:0/18:0) were abundant species in PI and PS, respectively. Twenty types of SM were detected; the most abundant types were SM (d18:1/22:0), SM (d18:1/16:0), and SM (18:1/23:0). Similar to that in the neutral lipids, 18:1, 18:2, 16:0, and 18:0 were also the abundant FAs in phospholipid digestion products.

### Particle Size Distribution and Zeta-Potential Changes

**Figure 6:**
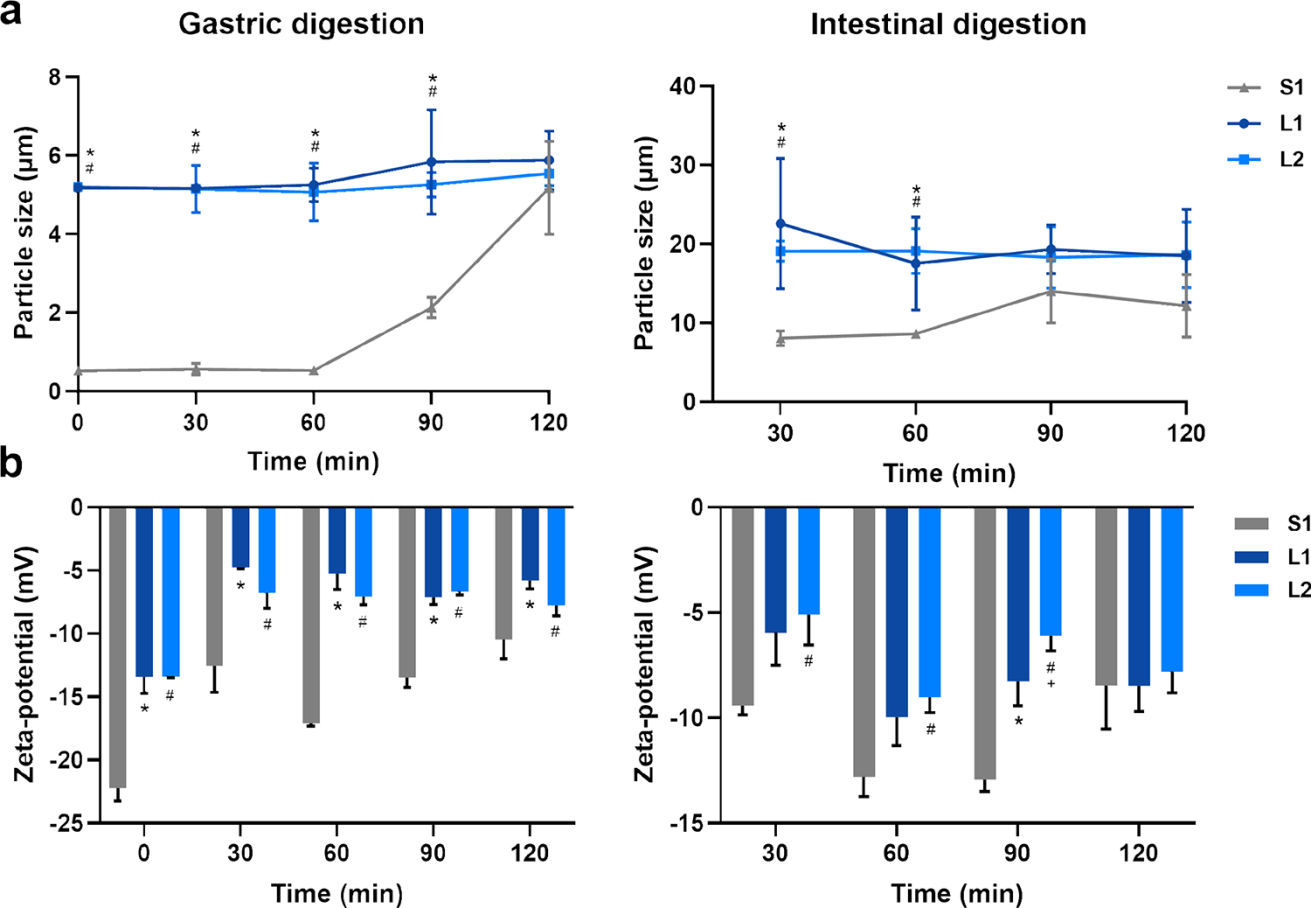
Structural changes of control milk formula and concept milk formulas during digestion. (a, b) Mean volume diameter (*D*_4,3_, μm) of lipid droplets in formula during (a) *in vitro* gastric and (b) intestinal digestion. (c, d) Zeta-potential (mV) of lipid droplets in formula during (c) *in vitro* gastric and (d) intestinal digestion. S1, gray triangle solid up; L1, dark-blue circle solid; and L2, light-blue box solid. The statistical analyses for each experiment are shown in the figures. *, ^#^, and ^+^ represented significant difference between S1 and L1, S1 and L2, and L1 and L2, respectively (*P* < 0.05).

The mean particle size and distribution of milk formulas during *in vitro* gastrointestinal digestion are shown in Figure 6a and Figure S1, respectively. Differences in degree of aggregation and/or dispersion could be observed through changes in particle size during *in vitro* digestion. As the gastric digestion progressed, the *D*_4,3_ of the milk formulas had a rising trend from G60, in which S1 kept the significantly lower mean particle size than L1 or L2 until G90 as was the case initially (*P* < 0.05). The proportion of over 3 μm in aggregates of S1 gradually increased from G90, as a result of its lipid droplets aggregation. The hydrolysis of the proteins around the lipid droplets by gastric pepsin resulted in the loss of the surface charge of the lipid droplets and thinning of the adsorption layer, resulting in an unstable system where the droplets tend to coalescence.^17^ The *D*_4,3_ of L1 and L2 showed a slow increase, and the particle size distribution range (0.6–30 μm) displayed no considerable change during the gastric phase. The *D*_4,3_ of S1 increased to 8 μm, while the *D*_4,3_ of L1 or L2 increased to 20 μm once the chyme was delivered into the intestinal phase, showing significantly lower mean particle size in S1 until I60 (*P* < 0.05). The particle size distribution of the milk formulas displayed a bimodal or trimodal profile during digestion, which could be attributed to the destruction of lipid droplets, which is consistent with previous reports.^44^ No significant difference in *D*_4,3_ was found between S1 and L1/L2 from I90, illustrating that the aggregates with digestion products included lipolysis and proteolysis products. This could also help explain why no significant difference in LDs was found at the end of the digestion.

The zeta-potential of the milk formulas during gastrointestinal digestion is shown in Figure 6b. During the gastric digestion, the absolute zeta-potential of the three milk formulas decreased because of salt ions in simulated digestive fluids generating electrostatic shielding, resulting in an increase in zeta-potential.^45^ The absolute value of zeta-potential in S1 was higher (*P* < 0.05) than L1/L2 throughout digestion, indicating their different degrees of protein (aggregates) on the interface. Lipid droplets in L1/L2 covered by MFGM components could adsorb the lipase through the interface directly, while proteins on the interface of S1 need be hydrolyzed first. In the intestinal phase, the absolute zeta-potential increased due to the presence of bile salts, which could replace the digestion products, including DAGs, MAGs, and FFAs, whose complex colloidal structures would leave the surface of droplets negatively charged.^17^

### Structural Changes of Milk Formulas during *In Vitro* Digestion

**Figure 7:**
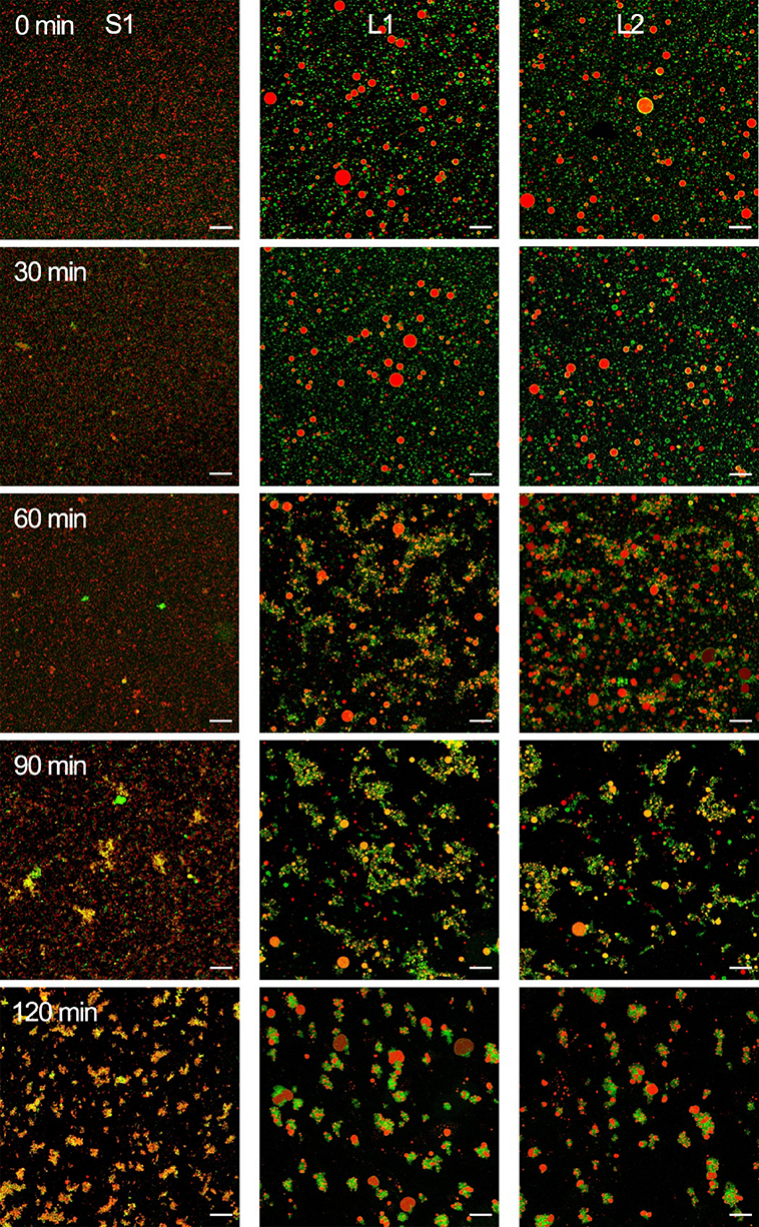
Microstructure changes of lipid droplets in milk formulas at 0, 30, 60, 90, and 120 min during *in vitro* gastric digestion observed by CLSM. Lipid droplets double-stained with Nile Red (red) and NBD-PC (green) fluorescent probes. All scale bars, 20 μm.

**Figure 8:**
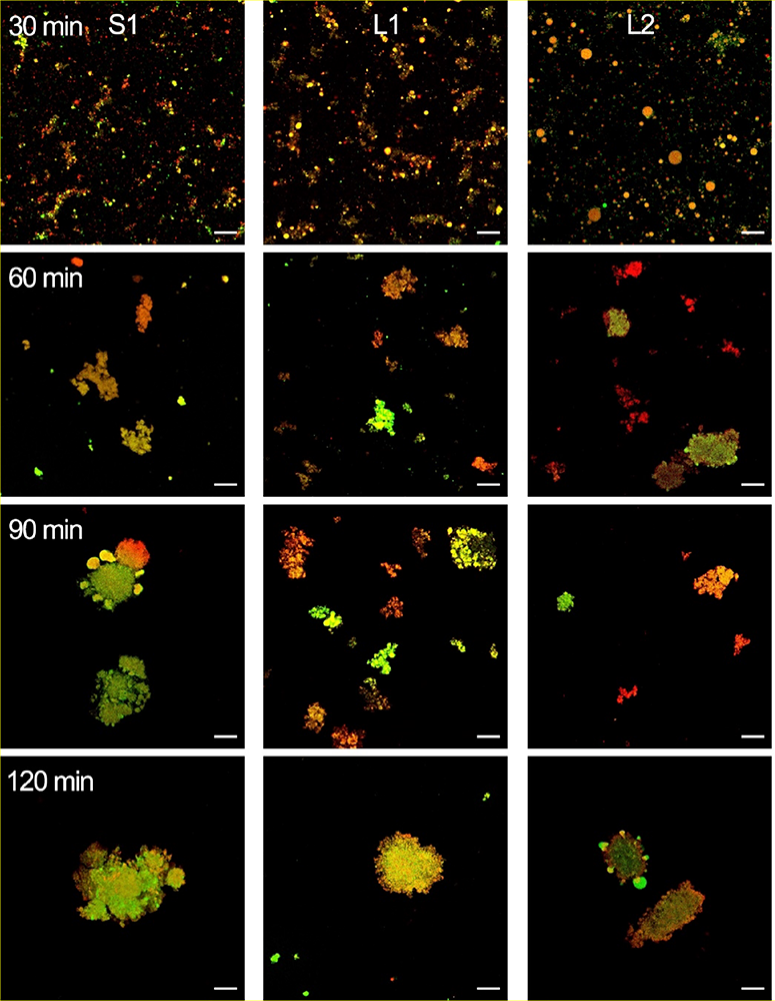
Microstructure changes of lipid droplets in milk formulas at 0, 30, 60, 90, and 120 min during *in vitro* intestinal digestion observed by CLSM. Lipid droplets double-stained with Nile Red (red) and NBD-PC (green) fluorescent probes. All scale bars, 20 μm.

The CLSM images of S1, L1, and L2 during *in vitro* gastrointestinal digestion are shown in Figures 7 and 8 and Figures S2 and S3. The initial lipid droplet structure covered with phospholipids in L1 and L2 could still be observed at 30 min gastric digestion, may due to gastric lipase hydrolyzed the TAGs within lipid droplets without inhibiting membrane.^31^ From G60, lipid droplets start to aggregate, with this process becoming much more pronounced as gastric digestion progressed. Phospholipids and membrane proteins were detached from lipid droplets, fused, and then scattered into the emulsion matrix in L1 and L2. From G90, phospholipids and membrane proteins aggregated evidently in S1, indicating the morphological instability of MFGM ingredients added in milk formulas during the digestion. The particle sizes observed in CLSM were consistent with the results above (Figure 6).

During the intestinal phase, fewer lipid droplets were observed, revealing the activity of pancreatin. At I60, both proteolysis and lipolysis products covered the droplets, increasing their particle size and decreasing their SSA. L1 and L2 are closer to the structural characteristics reported for natural milk fat during *in vitro* gastrointestinal digestion.^27^ At the end of the entire digestion, few but large aggregates were observed, in which polar lipids were gathered with the remaining fats. The mixed aggregates of apolar and amphiphilic compounds displayed in the CLSM images during the intestinal phase are likely to be particle aggregates.

**Figure 9:**
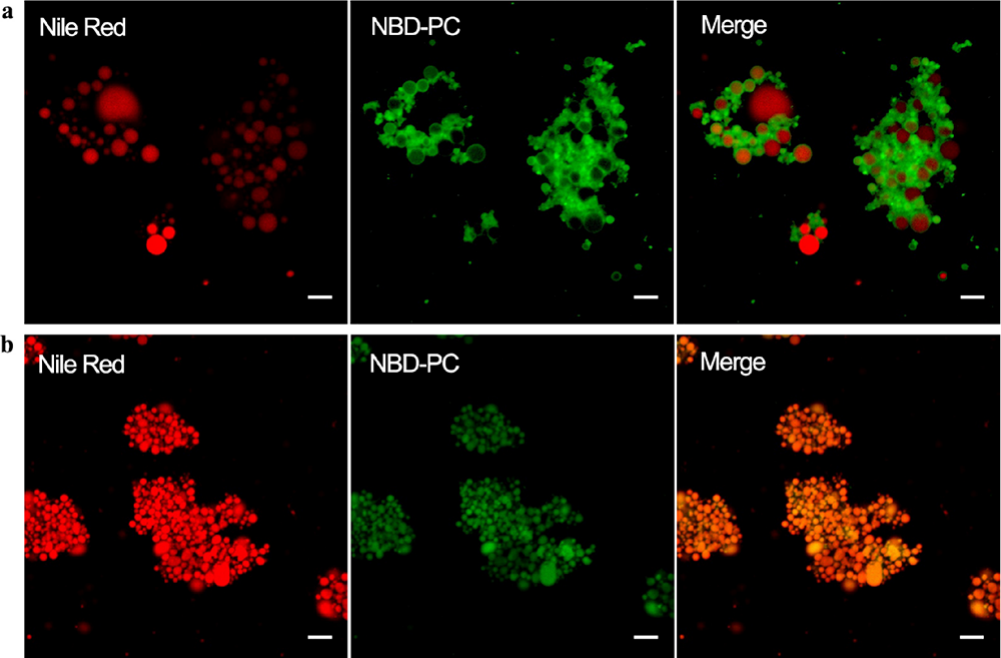
Fluorescent images observed by the CLSM demonstrating gastric emulsion microstructure (G120) of lipid droplets in L1 (a) and S1 (b). Left panel, red, neutral lipids stained by Nile Red; middle panel, green, phosphatidylcholine stained by NBD-PC; right panel, the two (left and middle) merged CLSM images. All scale bars, 5 μm.

In this study, we observed the gastric emulsion structure at the end of the gastric phase by CLSM, as shown in Figure 9. There were obvious structural differences between concept and control milk formulas. Concept milk formula (L1) showed an almost complete spherical structure with a mean size of 3–5 μm, which is consistent with data analyzed by laser light scattering, while control milk formula S1 showed large aggregates consisting of small lipid droplets, which is similar to Figure 7. Phospholipids, proteins, and TAGs are cross-linked together, and the contact area became smaller in subsequent intestinal digestion. The structure of lipid droplets formed in the gastric phase (shape, size, SSA, interface, etc.) influences the initial digestion in the intestinal phase. Studies reported that the droplet structure is a critical factor regulating the gastric lipolysis rate, the LD of complete gastric lipolysis, and the fatty acid release pattern.^46^ A similar gastric digestion emulsion structure was also observed in milk formulas with large droplets^44^ and in human milk^31^ digestion. The gastric emulsion structure has been shown to influence the gastric emulsion stability,^19,47^ further affect gastric emptying and lipid release.^48,49^ Detailed structural characterization of the gastric emulsion structure in milk formulas during digestion, especially *in vivo*, should be studied in the future.

In summary, the digestion profiles of milk formulas with different lipid droplet structures and compositions were evaluated using a dynamic *in vitro* digestion model simulating infant gastrointestinal digestion. Concept formulas L1 and L2 with different lipid sources had a mean particle size (*D*_4,3_ ∼5 μm) similar to human milk, with a core of triacylglycerols that were coated with phospholipids from MFGM ingredient. Control formula S1 contained the same lipid sources as L1, but the particle size was smaller (*D*_4,3_ ∼0.5 μm), which is similar to conventional milk formulas, and more protein was observed to be present on the interfaces of the lipid droplets of S1 compared to the concept formulas. During digestion, the lipolysis of L1 and L2 was slower than S1 during the gastric phase and up to I60 in the intestinal phase but was faster starting from I90 in the intestinal phase. No significant differences were found in LD at the end of the entire digestion between these milk formulas. Similar results were observed in the kinetic model in which the time point rate (*r*) of S1 was smaller than that of L1 or L2 after I60. The species of digestion products was highly related to their lipid sources, and the lipolysis rates were affected by the lipid droplet structure. Approximately 60–80% of glycerophospholipids were hydrolyzed to lysophospholipids, while SMs were barely hydrolyzed. At the end of the gastric digestion phase, concept formulas showed lipid droplet structures mostly remained intact, while control formula had larger aggregates of small lipid droplets. Differences in the structure of gastric digestion emulsion of milk formulas affected lipid release during the intestinal digestion phase. This study demonstrates the influences of the lipid droplet structure during gastric digestion and its consequences for intestinal digestion. This may provide a scientific rationale for further studies on milk formulas that aim to mimic natural milk fat globules to better deliver health benefits.

## Supporting Information Available

(Tables S1–S3) The fatty acid profile, *sn*-2 fatty acids profile, and major triacylglycerol compositions of milk formulas; (Tables S4–S6) the relative content lipolysis products control milk formula S1, concept milk formula L1, and L2 during *in vitro* digestion; (Table S7) concentration of phospholipid lipolysis products molecular species in milk formulas during *in vitro* digestion; and (Figures S1 and S2) CLSM images of S1, L1, and L2 at 0, 30, 60, 90, and 120 min during *in vitro* gastric and intestinal digestion (PDF)

## Abbreviations Used

CLSM: confocal laser scanning microscope
DAG: diacylglycerol
FFA: free fatty acid
MFG: milk fat globule
LD: lipolysis degree
LPC: lysophosphatidylcholine
LPE: lysophosphatidylethanolamine
MAG: monoacylglycerol
MFGM ingredients: milk fat globule membrane ingredients extraction from bovine milk
PC: phosphatidylcholine
PE: phosphatidylethanolamine
PI: phosphatidylinositol
PS: phosphatidylserine
Q-TOF-MS: quadrupole time-of-flight mass spectrometry
RGE: rabbit gastric extract
SSA: specific spread area
SGF: simulated gastric fluid
SIF: simulated intestinal fluid
SM: sphingomyelin
TAG: triacylglycerol
TEM: transmission electron microscope
UPLC: ultraperformance liquid chromatography

## References

[ref1] Jensen R. G. The lipids in human milk. Prog. Lipid Res. 1996, 35 (1), 53–92. 10.1016/0163-7827(95)00010-0. 9039426

[ref2] Roelofs J. J. M.; Tjoelker R. S.; Lambers T. T.; Smeets P. A. M. Mild processing and addition of milk fat globule membrane in infant formula may better mimic intragastric behavior of human milk: A proof of concept trial in healthy males. Food Hydrocolloids 2024, 151, 109839 10.1016/j.foodhyd.2024.109839.

[ref3] Gallier S.; Vocking K.; Post J. A.; Van De Heijning B.; Acton D.; Van Der Beek E. M.; Van Baalen T. A novel infant milk formula concept: Mimicking the human milk fat globule structure. Colloids Surf. B Biointerfaces 2015, 136, 329–39. 10.1016/j.colsurfb.2015.09.024. 26432620

[ref4] Lopez C.; Menard O. Human milk fat globules: polar lipid composition and in situ structural investigations revealing the heterogeneous distribution of proteins and the lateral segregation of sphingomyelin in the biological membrane. Colloids Surf., B 2011, 83 (1), 29–41. 10.1016/j.colsurfb.2010.10.039. 21126862

[ref5] Zou L.; Pande G.; Akoh C. C. Infant Formula Fat Analogs and Human Milk Fat: New Focus on Infant Developmental Needs. Annu. Rev. Food Sci. Technol. 2016, 7, 139–65. 10.1146/annurev-food-041715-033120. 26934172

[ref6] Pan Y.; Liu L.; Tian S.; Li X.; Hussain M.; Li C.; Zhang L.; Zhang Q.; Leng Y.; Jiang S.; Liang S. Comparative analysis of interfacial composition and structure of fat globules in human milk and infant formulas. Food Hydrocolloids 2022, 124, 107290 10.1016/j.foodhyd.2021.107290.

[ref7] Sun Y.; Ma S.; Liu Y.; Jia Z.; Li X.; Liu L.; Ma Q.; Jean Eric-parfait Kouame K.; Li C.; Leng Y.; Jiang S. Changes in interfacial composition and structure of milk fat globules are crucial regulating lipid digestion in simulated in-vitro infant gastrointestinal digestion. Food Hydrocolloids 2023, 134, 108003 10.1016/j.foodhyd.2022.108003.

[ref8] Oosting A.; Harvey L.; Ringler S.; van Dijk G.; Schipper L. Beyond ingredients: Supramolecular structure of lipid droplets in infant formula affects metabolic and brain function in mouse models. PLoS One 2023, 18 (8), e0282816 10.1371/journal.pone.0282816. 37531323 PMC10395839

[ref9] Baars A.; Oosting A.; Engels E.; Kegler D.; Kodde A.; Schipper L.; Verkade H. J.; van der Beek E. M. Milk fat globule membrane coating of large lipid droplets in the diet of young mice prevents body fat accumulation in adulthood. Br. J. Nutr. 2016, 115 (11), 1930–1937. 10.1017/S0007114516001082. 27040581 PMC4863696

[ref10] Jelenik T.; Kodde A.; Pesta D.; Phielix E.; Oosting A.; Rohbeck E.; Dewidar B.; Mastrototaro L.; Trenkamp S.; Keijer J.; van der Beek E. M.; Roden M. Dietary lipid droplet structure in postnatal life improves hepatic energy and lipid metabolism in a mouse model for postnatal programming. Pharmacol. Res. 2022, 179, 106193 10.1016/j.phrs.2022.106193. 35358682

[ref11] Teoh O. H.; Lin T. P.; Abrahamse-Berkeveld M.; Winokan A.; Chong Y. S.; Yap F.; Marintcheva-Petrova M.; van der Beek E. M.; Shek L. P. An Infant Formula with Large, Milk Phospholipid-Coated Lipid Droplets Supports Adequate Growth and Is Well-Tolerated in Healthy, Term Asian Infants: A Randomized, Controlled Double-Blind Clinical Trial. Nutrients 2022, 14 (3), 634 10.3390/nu14030634. 35276993 PMC8838783

[ref12] Breij L. M.; Abrahamse-Berkeveld M.; Vandenplas Y.; Jespers S. N. J.; De Mol A. C.; Khoo P. C.; Kalenga M.; Peeters S.; Van Beek R. H. T.; Norbruis O. F.; Schoen S.; Acton D.; Hokken-Koelega A. C. S.; An infant formula with large, milk phospholipid-coated lipid droplets containing a mixture of dairy and vegetable lipids supports adequate growth and is well tolerated in healthy, term infants. Am. J. Clin. Nutr. 2019, 109 (3), 586–596. 10.1093/ajcn/nqy322. 30793165 PMC6408203

[ref13] Abrahamse-Berkeveld M.; Jespers S. N.; Khoo P. C.; Rigo V.; Peeters S. M.; Van Beek R. H.; Norbruis O. F.; Schoen S.; Marintcheva-Petrova M.; Van der Beek E. M.; Stoelhorst G. M.; Vandenplas Y.; Hokken-Koelega A. C.; Infant Milk Formula with Large, Milk Phospholipid-coated Lipid Droplets Enriched in Dairy Lipids Affects Body Mass Index Trajectories and Blood Pressure at School Age: Follow-up of a Randomized Controlled Trial. Am. J. Clin. Nutr. 2024, 119 (1), 87–99. 10.1016/j.ajcnut.2023.10.017. 37973475

[ref14] Schipper L.; Bartke N.; Marintcheva-Petrova M.; Schoen S.; Vandenplas Y.; Hokken-Koelega A. C. S. Infant formula containing large, milk phospholipid-coated lipid droplets and dairy lipids affects cognitive performance at school age. Front. Nutr. 2023, 10, 1215199 10.3389/fnut.2023.1215199. 37731397 PMC10508340

[ref15] Thomassen G. G. M.; Abrahamse E.; Mischke M.; Becker M.; Bartke N.; Knol J.; Renes I. B. In vitro gastrointestinal lipid handling and bioaccessibility rate of infant formula with large phospholipid-coated lipid droplets are different from those of standard formula and closer to human milk. Food Hydrocolloids 2024, 156, 110336 10.1016/j.foodhyd.2024.110336.

[ref16] Wang L.; Zhang X.; Yuan T.; Jin Q.; Wei W.; Wang X. Digestion of medium- and long-chain triacylglycerol and sn-2 palmitate in infant formula: A study based on dynamic in vitro simulation of infant gastrointestinal lipolysis. J. Agric. Food. Chem. 2022, 70 (10), 3263–3271. 10.1021/acs.jafc.1c07118. 35255218

[ref17] Liu L.; Zhang X.; Liu Y.; Wang L.; Li X. Simulated In Vitro Infant Gastrointestinal Digestion of Infant Formulas Containing Different Fat Sources and Human Milk: Differences in Lipid Profiling and Free Fatty Acid Release. J. Agric. Food Chem. 2021, 69 (24), 6799–6809. 10.1021/acs.jafc.1c01760. 34126744

[ref18] Infantes-Garcia M. R.; Verkempinck S. H. E.; Guevara-Zambrano J. M.; Andreoletti C.; Hendrickx M. E.; Grauwet T. Enzymatic and chemical conversions taking place during in vitro gastric lipid digestion: The effect of emulsion droplet size behavior. Food Chem. 2020, 326, 126895 10.1016/j.foodchem.2020.126895. 32438227

[ref19] Infantes-Garcia M. R.; Verkempinck S. H. E.; Del Castillo-Santaella T.; Maldonado-Valderrama J.; Hendrickx M. E.; Grauwet T. In vitro gastric lipid digestion of emulsions with mixed emulsifiers: Correlation between lipolysis kinetics and interfacial characteristics. Food Hydrocolloids 2022, 128, 107576 10.1016/j.foodhyd.2022.107576.

[ref20] Luo J.; Wang Z.; Li Y.; Chen C.; Ren F.; Guo H. The simulated in vitro infant gastrointestinal digestion of droplets covered with milk fat globule membrane polar lipids concentrate. J. Dairy Sci. 2018, 102, 2879–2889. 10.3168/jds.2018-15044. 30738688

[ref21] Folch J.; Lees M.; Stanley G. H. S. A simple method for the isolation and purification of total lipids from animal Tissues. J. Biol. Chem. 1957, 226 (1), 497–509. 10.1016/S0021-9258(18)64849-5. 13428781

[ref22] Zhang X.; Wei W.; Tao G.; Jin Q.; Wang X. Identification and Quantification of Triacylglycerols Using Ultraperformance Supercritical Fluid Chromatography and Quadrupole Time-of-Flight Mass Spectrometry: Comparison of Human Milk, Infant Formula, Other Mammalian Milk, and Plant Oil. J. Agric. Food Chem. 2021, 69 (32), 8991–9003. 10.1021/acs.jafc.0c07312. 33755452

[ref23] Qi C.; Sun J.; Xia Y.; Yu R.; Wei W.; Xiang J.; Jin Q.; Xiao H.; Wang X. Fatty acid profile and the sn-2 position distribution in triacylglycerols of breast milk during different lactation stages. J. Agric. Food Chem. 2018, 66 (12), 3118–3126. 10.1021/acs.jafc.8b01085. 29526089

[ref24] Yu J.; Wei W.; Wang F.; Yu R.; Jin Q.; Wang X. Evaluation of total, sn-2 fatty acid, and triacylglycerol composition in commercial infant formulas on the Chinese market: A comparative study of preterm and term formulas. Food Chem. 2022, 384, 132477 10.1016/j.foodchem.2022.132477. 35219236

[ref25] Wei W.; Yang J.; Yang D.; Wang X.; Yang Z.; Jin Q.; Wang M.; Lai J.; Wang X. Phospholipid Composition and Fat Globule Structure I: Comparison of Human Milk Fat from Different Gestational Ages, Lactation Stages, and Infant Formulas. J. Agric. Food Chem. 2019, 67 (50), 13922–13928. 10.1021/acs.jafc.9b04247. 31746600

[ref26] Jiang C.; Zhang X.; Yu J.; Yuan T.; Zhao P.; Tao G.; Wei W.; Wang X. Comprehensive lipidomic analysis of milk polar lipids using ultraperformance supercritical fluid chromatography-mass spectrometry. Food Chem. 2022, 393, 133336 10.1016/j.foodchem.2022.133336. 35691069

[ref27] Zhao P.; Yang X.; Li D.; Zhang X.; Wei W.; Jin Q.; Wang X. Development of in vitro digestion simulation of gastrointestinal tract to evaluate lipolysis and proteolysis: Comparison of infant model digestion of breast milk and adult model digestion of cow milk. Food Hydrocolloids 2023, 142, 108859 10.1016/j.foodhyd.2023.108859.

[ref28] Infantes-Garcia M. R.; Verkempinck S. H. E.; Hendrickx M. E.; Grauwet T. Kinetic Modeling of In Vitro Small Intestinal Lipid Digestion as Affected by the Emulsion Interfacial Composition and Gastric Prelipolysis. J. Agric. Food Chem. 2021, 69 (16), 4708–4719. 10.1021/acs.jafc.1c00432. 33856215

[ref29] Zhao P.; Li D.; Zhang X.; Ye X.; Zhang Z.; Liu Z.; Yan Z.; Wei W.; Jin Q.; Wang X. Comparison of lipid structure and composition in human or cow’s milk with different fat globules by homogenization. Food Funct. 2023, 14 (12), 5631–5643. 10.1039/D2FO02515A. 37233209

[ref30] Weng J.; Lin R.; Jiang C.; Wei W.; Wang X.; Jin Q. O/W Emulsion Stabilized by Bovine Milk Phospholipid-Protein Nanoemulsions: Preparation, Stability, and In Vitro Digestion. J. Agric. Food Chem. 2021, 69 (17), 5003–5012. 10.1021/acs.jafc.0c05617. 33886291

[ref31] Wei W.; Yang X.; Zhao P.; Gan J.; Abrahamse E.; Bartke N.; Zhao X.; Wang X. Structural changes and triacylglycerol lipolysis products of milk formula with large phospholipid-coated lipid droplets during in vitro digestion: Comparison with human milk and commercial standard formulas. Food Hydrocolloids 2024, 151, 109831 10.1016/j.foodhyd.2024.109831.

[ref32] Karakus M. S.; Akgul F. Y.; Korkmaz A.; Atasoy A. F. Evaluation of fatty acids, free fatty acids and textural properties of butter and sadeyag (anhydrous butter fat) produced from ovine and bovine cream and yoghurt. Int. Dairy J. 2022, 126, 105229 10.1016/j.idairyj.2021.105229.

[ref33] Zhao P.; Zhang S.; Liu L.; Pang X.; Yang Y.; Lu J.; Lv J. Differences in the Triacylglycerol and Fatty Acid Compositions of Human Colostrum and Mature Milk. J. Agric. Food Chem. 2018, 66 (17), 4571–4579. 10.1021/acs.jafc.8b00868. 29658706

[ref34] Sun C.; Wei W.; Su H.; Zou X.; Wang X. Evaluation of sn-2 fatty acid composition in commercial infant formulas on the Chinese market: A comparative study based on fat source and stage. Food Chem. 2018, 242, 29–36. 10.1016/j.foodchem.2017.09.005. 29037692

[ref35] Tu A.; Ma Q.; Bai H.; Du Z. A comparative study of triacylglycerol composition in Chinese human milk within different lactation stages and imported infant formula by SFC coupled with Q-TOF-MS. Food Chem. 2017, 221, 555–567. 10.1016/j.foodchem.2016.11.139. 27979241

[ref36] Song S.; Liu T. T.; Liang X.; Liu Z. Y.; Yishake D.; Lu X. T.; Yang M. T.; Man Q. Q.; Zhang J.; Zhu H. L. Profiling of phospholipid molecular species in human breast milk of Chinese mothers and comprehensive analysis of phospholipidomic characteristics at different lactation stages. Food Chem. 2021, 348, 129091 10.1016/j.foodchem.2021.129091. 33508603

[ref37] Wei W.; Li D.; Jiang C.; Zhang X.; Zhang X.; Jin Q.; Zhang X.; Wang X. Phospholipid composition and fat globule structure II: Comparison of mammalian milk from five different species. Food Chem. 2022, 388, 132939 10.1016/j.foodchem.2022.132939. 35447582

[ref38] Ma Q.; Sun M.; Zhao Y.; Chen S.; Li X.; Liu L.; Zhang X.; Wang Y.; Eric-Parfait Kouame K. J.; Yu X. Improving lipid digestion by modulating interfacial structure of fat globule based on milk fat globule membrane and different phospholipids. Food Hydrocolloids 2024, 150, 109736 10.1016/j.foodhyd.2024.109736.

[ref39] Pafumi Y.; Lairon D.; de la Porte P. L.; Juhel C.; Storch J.; Hamosh M.; Armand M. Mechanisms of inhibition of triacylglycerol hydrolysis by human gastric lipase. J. Biol. Chem. 2002, 277 (31), 28070–9. 10.1074/jbc.M202839200. 11940604

[ref40] Wang Y.; Xu H.; Liu X.; Wei W.; Jin Q.; Wang X. The structure of triglycerides impacts the digestibility and bioaccessibility of nutritional lipids during in vitro simulated digestion. Food Chem. 2023, 418, 135947 10.1016/j.foodchem.2023.135947. 36996650

[ref41] Teng F.; Reis M. G.; Yang L.; Ma Y.; Day L. Structural characteristics of triacylglycerols contribute to the distinct in vitro gastric digestibility of sheep and cow milk fat prior to and after homogenisation. Food Res. Int. 2020, 130, 108911 10.1016/j.foodres.2019.108911. 32156362

[ref42] Nilsson A.; Duan R. D.; Ohlsson L. Digestion and Absorption of Milk Phospholipids in Newborns and Adults. Front. Nutr. 2021, 8, 724006 10.3389/fnut.2021.724006. 34490332 PMC8417471

[ref43] Aloulou A.; Frikha F.; Noiriel A.; Bou Ali M.; Abousalham A. Kinetic and structural characterization of triacylglycerol lipases possessing phospholipase A1 activity. Biochim. Biophys. Acta 2014, 1841 (4), 581–587. 10.1016/j.bbalip.2013.12.009. 24368210

[ref44] Bourlieu C.; Menard O.; De La Chevasnerie A.; Sams L.; Rousseau F.; Madec M. N.; Robert B.; Deglaire A.; Pezennec S.; Bouhallab S.; Carrière F.; Dupont D. The structure of infant formulas impacts their lipolysis, proteolysis and disintegration during in vitro gastric digestion. Food Chem. 2015, 182, 224–235. 10.1016/j.foodchem.2015.03.001. 25842331

[ref45] Pan Y.; Liu S.; Li J.; Hussain M.; Bora A. F. M.; Li X.; Liu L.; Liu W.; Li L.; Zhu B.; Zhou W. Regulating the lipid droplet interface based on milk fat globule membrane and milk proteins to improve lipid digestion of model infant formula emulsion. Food Hydrocolloids 2024, 146, 109187 10.1016/j.foodhyd.2023.109187.

[ref46] Ma Q.; Zhang X.; Li X.; Liu L.; Liu S.; Hao D.; Bora A. F. M.; Kouame K. J. E. P.; Xu Y.; Liu W.; Li J. Novel trends and challenges in fat modification of next-generation infant formula: Considering the structure of milk fat globules to improve lipid digestion and metabolism of infants. Food Res. Int. 2023, 174 (Pt 1), 113574 10.1016/j.foodres.2023.113574. 37986523

[ref47] Scheuble N.; Schaffner J.; Schumacher M.; Windhab E. J.; Liu D.; Parker H.; Steingoetter A.; Fischer P. Tailoring Emulsions for Controlled Lipid Release: Establishing in vitro–in Vivo Correlation for Digestion of Lipids. ACS Appl. Mater. Interfaces 2018, 10 (21), 17571–17581. 10.1021/acsami.8b02637. 29708724

[ref48] Infantes-Garcia M. R.; Verkempinck S. H. E.; Gonzalez-Fuentes P. G.; Hendrickx M. E.; Grauwet T. Lipolysis products formation during in vitro gastric digestion is affected by the emulsion interfacial composition. Food Hydrocolloids 2021, 110, 106163 10.1016/j.foodhyd.2020.106163.

[ref49] Milan A. M.; Barnett M. P. G.; McNabb W. C.; Roy N. C.; Coutinho S.; Hoad C. L.; Marciani L.; Nivins S.; Sharif H.; Calder S.; Du P.; Gharibans A. A.; O’Grady G.; Fraser K.; Bernstein D.; Rosanowski S. M.; Sharma P.; Shrestha A.; Mithen R. F. The impact of heat treatment of bovine milk on gastric emptying and nutrient appearance in peripheral circulation in healthy females: a randomized controlled trial comparing pasteurized and ultra-high temperature milk. American Journal of Clinical Nutrition 2024, 119 (5), 1200–1215. 10.1016/j.ajcnut.2024.03.002. 38452857

