# Supplementary material for: In Vitro Lipid Digestion of Milk Formula with Different Lipid Droplets: A Study on the Gastric Digestion Emulsion Structure and Lipid Release Pattern
Source: J Agric Food Chem. 2024 Oct 28;72(44):24736–48. doi: 10.1021/acs.jafc.4c05114 (PMC11544712; doi:10.1021/acs.jafc.4c05114)
Supplement: Supplementary file 1 — jf4c05114_si_001.pdf [file jf4c05114_si_001.pdf]

Supporting Information

In vitro Lipid Digestion of Milk Formula with Different Lipid Droplets: A Study on Gastric Digestion Emulsion Structure and Lipid Release Pattern

Pu Zhao, Xue Yang, Junai Gan, Ingrid Renes, Evan Abrahamse, Nana Bartke,

Wei Wei*, and Xingguo Wang

Table S1. The fatty acid profile (wt%) of milk formulas. Page 2

Table S2. The *sn*-2 fatty acids profile (wt%) of milk formulas. Page 3

Table S3. The major triacylglycerol compositions (wt%) of milk formulas. Page 4

Table S4. The relative content (%) of lipolysis products (DAGs, MAGs, and FFAs) and undigested TAGs in control milk formula S1 during in vitro digestion. Page 5

Table S5. The relative content (%) of lipolysis products (DAGs, MAGs, and FFAs) and undigested TAGs in concept milk formula L1 during in vitro digestion. Page 7

Table S6. The relative content (%) of lipolysis products (DAGs, MAGs, and FFAs) and undigested TAGs in concept milk formula L2 during in vitro digestion. Page 9

Table S7. Concentration of phospholipid lipolysis products molecular species (μmol/g fat) in S1, L1, and L2 during in vitro digestion. Page 11

Figure S1. CLSM images of S1, L1, and L2 at 0, 30, 60, 90, and 120 min during in vitro gastric digestion. Neutral lipids strained with Nile Red (red), and glycoproteins & glycolipids stained with WGA (blue). All scale bar, 20 μm. Page 15

Figure S2. CLSM images of S1, L1, and L2 at 0, 30, 60, 90, and 120 min during in vitro intestinal digestion. Neutral lipids strained with Nile Red (red), and glycoproteins & glycolipids stained with WGA (blue). All scale bar, 20 μm. Page 16

Table S1. The fatty acid profile (wt%) of milk formulas.

Fatty acids	S1	L1	L2
SFA	42.05 ± 0.36	42.03 ± 0.32	42.98 ± 1.08
MUFA	39.77 ± 0.24	39.69 ± 0.20	38.14 ± 0.70
PUFA	18.18 ± 0.17	18.27 ± 0.12	18.87 ± 0.36
n-6/ n-3 PUFA	6.40 ± 0.16	6.49 ± 0.07	6.32 ± 0.05
LA/ALA	8.53 ± 0.17	8.24 ± 0.16	8.63 ± 0.09

The values are represented as the means ± SD; SFA, saturated fatty acid; MUFA, monounsaturated fatty acid; PUFA, polyunsaturated fatty acid; LA, linoleic acid; ALA, α-linolenic Acid.

Table S2. The *sn*-2 fatty acids profile (wt%) of milk formulas.

Fatty acids	S1	L1	L2
SFAs	24.70 ± 1.83^{b}	25.07 ± 2.61^{b}	38.26 ± 4.31^{a}
MUFAs	50.73 ± 3.02	49.03 ± 4.00	40.35 ± 5.57
PUFAs	24.59 ± 1.20^{b}	25.89 ± 1.51^{a}	21.41 ± 1.35^{b}
n-6/ n-3 PUFA	6.82 ± 0.27	7.10 ± 0.04	6.67 ± 0.33
LA/ALA	8.06 ± 0.18	8.02 ± 0.18	7.92 ± 0.41

The values are represented as the means ± SD; SFA, saturated fatty acid; MUFA, monounsaturated fatty acid; PUFA, polyunsaturated fatty acid; LA, linoleic acid; ALA, α-linolenic Acid. Different letters (a-b) in the row indicate significant ($P < 0.05$) differences among samples.

Table S3. The major triacylglycerol compositions (wt%) of milk formulas.

Triacylglycerols	S1	L1	L2
12:0/12:0/8:0	3.15 ± 0.12^{a}	3.45 ± 0.14^{a}	1.33 ± 0.15^{b}
12:0/12:0/12:0	4.11 ± 0.41^{a}	3.37 ± 0.51^{a}	1.31 ± 0.09^{b}
14:0/12:0/12:0	3.39 ± 0.27^{a}	3.92 ± 0.23^{a}	1.63 ± 0.37^{b}
18:1/16:0/16:0	12.67 ± 0.07^{a}	12.65 ± 0.17^{a}	2.87 ± 0.06^{b}
18:2/18:2/16:0	1.99 ± 0.04^{b}	2.00 ± 0.04^{b}	2.32 ± 0.03^{a}
18:2/18:1/16:0	4.92 ± 0.12^{a}	4.84 ± 0.25^{a}	2.30 ± 0.11^{b}
18:1/18:1/16:0	12.58 ± 0.15^{a}	12.58 ± 0.19^{a}	5.04 ± 0.23^{b}
18:2/18:2/18:2	3.00 ± 0.03^{b}	3.00 ± 0.11^{b}	4.82 ± 0.13^{a}
18:2/18:2/18:1	4.43 ± 0.19^{b}	4.71 ± 0.26^{b}	7.61 ± 0.36^{a}
18:2/18:1/18:1	6.39 ± 0.14^{a}	5.73 ± 0.09^{b}	5.33 ± 0.36^{b}
18:1/18:1/18:1	15.3 ± 0.14^{b}	16.02 ± 0.40^{ab}	16.26 ± 0.27^{a}
Others	28.07 ± 1.23^{b}	27.73 ± 0.97^{b}	49.18 ± 2.87^{a}

The values are represented as the means ± SD; nd, not detected. Different letters (a-b) in the row indicate significant ($P < 0.05$) differences among samples.

Table S4. The relative content (%) of lipolysis products (DAGs, MAGs, and FFAs) and undigested TAGs in control milk formula S1 during in vitro digestion.

No.	m/z	Molecular species	G0	G30	G60	G90	G120	I30	I60	I90	I120
Main TAGs											
1	600.8	12:0/12:0/8:0	2.93	2.27	1.17	0.83	0.62	0.42	0.13	0.04	0.04
2	656.8	12:0/12:0/12:0	3.77	2.74	2.25	1.85	1.66	0.58	0.18	0.22	0.19
3	684.8	14:0/12:0/12:0	3.41	3.00	2.45	2.27	2.12	0.63	0.22	0.25	0.22
4	850.8	18:1/16:0/16:0	13.20	12.37	13.24	13.23	12.77	3.27	0.95	0.78	0.68
5	872.8	18:2/18:2/16:0	1.72	2.03	2.02	2.20	2.18	0.98	0.89	0.68	0.60
6	874.8	18:2/18:1/16:0	3.83	4.27	4.37	4.43	4.16	2.05	2.15	1.70	1.50
7	876.8	18:1/18:1/16:0	12.21	11.84	12.80	12.70	11.98	4.02	2.18	2.08	1.83
8	896.8	18:2/18:2/18:2	2.83	2.88	2.80	3.07	3.38	0.81	0.35	0.25	0.22
9	898.8	18:2/18:2/18:1	3.63	4.22	3.97	4.30	4.36	1.34	0.59	0.44	0.38
10	900.8	18:2/18:1/18:1	5.24	5.67	5.47	5.53	5.42	1.85	0.93	0.74	0.65
11	902.8	18:1/18:1/18:1	16.40	15.66	16.63	16.17	15.70	3.99	1.00	0.83	0.73
Others			31.72	30.78	28.47	27.16	26.82	12.68	10.56	8.97	7.88
Main DAGs											
1	479.5	12:0/12:0	0.14	0.31	0.32	0.43	0.31	0.23	0.12	0.19	0.17
2	507.5	14:0/12:0	0.13	0.36	0.36	0.50	0.41	0.14	0.17	0.12	0.07
3	591.5	16:0/16:0	0.41	0.35	0.30	0.34	0.36	0.56	0.61	0.91	0.33
4	615.5	18:2/16:0	0.21	0.17	0.12	0.14	0.13	0.25	0.22	0.28	0.17
5	617.5	18:1/16:0	0.28	0.26	0.21	0.25	0.23	0.36	0.26	0.34	0.23
6	619.5	18:0/16:0	0.78	0.47	0.54	0.60	0.65	1.35	1.52	2.05	0.82

7	639.5	18:2/18:2	0.12	0.09	0.08	0.09	0.08	0.42	0.45	0.49	0.28
8	641.5	18:2/18:1	0.14	0.12	0.10	0.13	0.13	0.90	1.17	1.39	1.01
9	643.5	18:1/18:1	0.32	0.22	0.18	0.20	0.18	0.81	1.01	1.15	0.87
Others			0.41	0.62	0.62	0.82	0.83	1.46	1.56	1.63	1.66
MAGs											
1	297.2	MAG-12:0	nd	nd	nd	nd	nd	0.70	1.12	0.89	0.82
2	325.2	MAG-14:0	nd	nd	nd	nd	nd	0.22	0.29	0.28	0.34
3	353.2	MAG-16:0	nd	nd	nd	nd	nd	0.82	1.23	1.02	2.59
4	375.2	MAG-18:3	nd	nd	nd	nd	nd	0.55	0.94	0.88	0.66
5	377.2	MAG-18:2	nd	nd	nd	nd	nd	0.90	1.58	1.42	1.67
6	379.2	MAG-18:1	nd	nd	nd	nd	nd	3.54	5.93	5.03	4.63
7	381.2	MAG-18:0	nd	nd	nd	nd	nd	1.06	1.11	1.38	2.47
8	401.2	MAG-20:4	nd	nd	nd	nd	nd	0.34	0.78	0.50	0.56
9	425.2	MAG-22:6	nd	nd	nd	nd	nd	0.70	1.25	1.08	0.76
10	427.2	MAG-22:5	nd	nd	nd	nd	nd	0.13	0.37	0.23	0.70
FFAs											
1	199.2	12:0	nd	nd	nd	nd	nd	4.79	4.79	4.78	4.90
2	227.2	14:0	nd	nd	nd	nd	nd	2.98	3.24	3.44	3.69
3	255.2	16:0	nd	0.94	2.18	2.28	2.75	12.02	13.76	15.30	15.91
4	277.2	18:3	nd	nd	nd	nd	nd	1.66	1.67	1.77	1.69
5	279.2	18:2	nd	nd	nd	nd	nd	8.00	8.78	8.73	8.88
6	281.2	18:1	nd	nd	nd	nd	1.26	16.46	18.37	19.05	19.94
7	283.2	18:0	nd	2.65	3.71	4.89	5.70	5.90	7.03	7.37	7.56
8	303.2	20:4	nd	nd	nd	nd	nd	1.07	1.28	1.35	1.57
9	327.2	22:6	nd	nd	nd	nd	nd	1.14	1.40	1.63	1.61

Table S5. The relative content (%) of lipolysis products (DAGs, MAGs, and FFAs) and undigested TAGs in concept milk formula L1 during in vitro digestion.

No.	m/z	Molecular species	G0	G30	G60	G90	G120	I30	I60	I90	I120
Main TAGs											
1	600.8	12:0/12:0/8:0	3.77	2.71	2.24	1.93	1.80	0.67	0.35	0.21	0.11
2	656.8	12:0/12:0/12:0	3.38	2.91	2.40	2.62	2.27	0.49	0.28	0.19	0.15
3	684.8	14:0/12:0/12:0	4.24	3.93	3.55	3.18	3.45	0.65	0.44	0.48	0.28
4	850.8	18:1/16:0/16:0	12.53	12.75	12.97	12.45	12.06	3.75	2.03	1.49	0.64
5	872.8	18:2/18:2/16:0	1.93	1.71	1.74	2.00	1.81	0.76	0.45	0.83	0.37
6	874.8	18:2/18:1/16:0	3.59	3.77	3.92	4.12	4.30	2.14	1.03	1.33	1.15
7	876.8	18:1/18:1/16:0	12.09	12.42	13.00	12.13	11.83	3.89	2.28	1.77	1.35
8	896.8	18:2/18:2/18:2	2.82	2.65	2.89	3.06	2.88	1.26	0.55	0.50	0.21
9	898.8	18:2/18:2/18:1	3.98	3.84	3.93	4.04	3.52	1.78	0.95	0.73	0.42
10	900.8	18:2/18:1/18:1	4.49	4.71	4.92	4.81	4.72	2.32	1.21	0.95	0.80
11	902.8	18:1/18:1/18:1	16.52	16.72	16.85	16.61	16.10	5.18	3.05	1.21	0.88
Others			29.19	27.14	24.07	24.11	24.16	8.39	5.69	7.03	5.12
Main DAGs											
1	423.5	12:0/8:0	0.02	0.03	0.03	0.04	0.04	0.45	0.39	0.18	0.07
2	479.5	12:0/12:0	0.05	0.24	0.26	0.43	0.44	0.87	0.84	0.37	0.13
3	507.5	14:0/12:0	0.07	0.26	0.40	0.41	0.46	0.65	0.41	0.15	0.07
4	591.5	16:0/16:0	0.20	0.23	0.33	0.29	0.30	0.63	0.49	0.67	0.29
5	615.5	18:2/16:0	0.13	0.16	0.16	0.20	0.21	0.90	0.96	0.60	0.19
6	617.5	18:1/16:0	0.16	0.22	0.24	0.31	0.31	1.13	1.40	0.87	0.26

7	619.5	18:0/16:0	0.40	0.33	0.52	0.48	0.49	1.58	1.42	1.46	0.63
8	639.5	18:2/18:2	0.05	0.04	0.07	0.06	0.07	1.43	0.63	0.24	0.21
9	641.5	18:2/18:1	0.07	0.09	0.10	0.13	0.14	1.92	2.13	1.81	0.77
10	643.5	18:1/18:1	0.18	0.20	0.22	0.28	0.29	1.83	1.52	1.73	0.69
Others			0.13	0.43	0.51	0.72	0.78	3.49	5.02	3.58	1.30
MAGs											
1	297.2	MAG-12:0	nd	nd	nd	nd	nd	1.20	1.08	0.43	1.28
2	325.2	MAG-14:0	nd	nd	nd	nd	nd	0.34	0.31	0.19	0.37
3	353.2	MAG-16:0	nd	nd	nd	nd	nd	1.28	0.80	1.17	1.61
4	375.2	MAG-18:3	nd	nd	nd	nd	nd	0.72	0.93	0.36	0.99
5	377.2	MAG-18:2	nd	nd	nd	nd	nd	1.61	1.93	1.02	2.18
6	379.2	MAG-18:1	nd	nd	nd	nd	nd	4.07	4.70	2.48	5.87
7	381.2	MAG-18:0	nd	nd	nd	nd	nd	1.14	0.68	1.04	1.77
8	401.2	MAG-20:4	nd	nd	nd	nd	nd	0.29	0.25	0.19	0.58
9	425.2	MAG-22:6	nd	nd	nd	nd	nd	0.55	0.67	0.32	1.06
10	427.2	MAG-22:5	nd	nd	nd	nd	nd	0.20	0.24	0.31	0.43
FFAs											
1	199.2	12:0	nd	nd	nd	nd	nd	3.06	4.63	5.71	5.69
2	227.2	14:0	nd	nd	nd	nd	nd	2.90	3.37	3.40	3.40
3	255.2	16:0	nd	0.90	1.48	2.35	3.67	10.05	11.65	14.23	15.65
4	277.2	18:3	nd	nd	nd	nd	nd	0.86	1.69	2.09	1.96
5	279.2	18:2	nd	nd	nd	nd	nd	7.85	8.29	9.24	10.32
6	281.2	18:1	nd	nd	nd	nd	nd	12.92	17.88	20.72	21.23
7	283.2	18:0	nd	1.63	3.21	3.25	3.94	3.56	5.32	5.80	6.32
8	303.2	20:4	nd	nd	nd	nd	nd	0.49	1.07	1.62	1.81
9	327.2	22:6	nd	nd	nd	nd	nd	0.73	1.03	1.42	1.39

Table S6. The relative content (%) of lipolysis products (DAGs, MAGs, and FFAs) and undigested TAGs in concept milk formula L2 during in vitro digestion.

No.	m/z	Molecular species	G0	G30	G60	G90	G120	I30	I60	I90	I120
Main TAGs											
1	600.8	12:0/12:0/8:0	1.35	1.00	0.91	0.93	0.91	0.19	0.13	0.11	0.01
2	656.8	12:0/12:0/12:0	1.31	1.17	0.99	1.09	1.07	0.26	0.18	0.11	0.01
3	684.8	14:0/12:0/12:0	1.58	1.45	1.36	1.29	1.27	0.30	0.22	0.12	0.02
4	850.8	18:1/16:0/16:0	3.28	3.21	3.46	3.25	3.20	2.85	2.95	1.85	1.71
5	872.8	18:2/18:2/16:0	1.96	1.98	2.11	2.08	2.04	2.18	1.00	0.55	0.91
6	874.8	18:2/18:1/16:0	2.20	2.10	2.04	1.98	1.95	0.78	0.84	0.83	0.77
7	876.8	18:1/18:1/16:0	5.06	4.88	5.18	4.92	4.84	3.29	2.00	1.32	1.25
8	896.8	18:2/18:2/18:2	4.03	4.58	4.21	4.24	4.17	1.59	0.59	0.30	0.44
9	898.8	18:2/18:2/18:1	6.54	6.12	6.86	6.38	6.28	4.55	2.60	1.82	1.65
10	900.8	18:2/18:1/18:1	4.87	4.73	4.51	4.42	4.35	2.66	1.41	0.68	0.97
11	902.8	18:1/18:1/18:1	15.12	15.23	15.95	14.63	14.39	3.92	1.81	0.99	0.83
Others			51.34	48.83	46.17	46.27	45.43	11.74	10.35	6.81	2.65
Main DAGs											
1	479.5	12:0/12:0	nd	0.04	0.06	0.11	0.11	0.21	0.06	0.05	0.04
2	507.5	14:0/12:0	0.06	0.07	0.12	0.15	0.15	0.17	0.08	0.05	0.05
3	591.5	16:0/16:0	0.16	0.13	0.21	0.33	0.33	0.66	0.42	0.40	0.28
4	615.5	18:2/16:0	0.13	0.06	0.08	0.14	0.23	0.51	0.39	0.25	0.29
5	619.5	18:0/16:0	0.28	0.20	0.30	0.50	0.45	1.34	0.95	0.86	0.57
6	639.5	18:2/18:2	0.12	0.07	0.11	0.17	0.31	0.95	0.61	0.26	0.54
7	641.5	18:2/18:1	0.07	0.04	0.05	0.08	0.31	1.20	0.92	0.55	0.76

8	643.5	18:1/18:1	0.19	0.10	0.13	0.22	0.25	1.02	0.58	0.37	0.41
9	647.5	18:0/18:0	0.19	0.42	0.56	0.94	0.86	3.28	2.56	3.43	2.28
Others			0.17	0.26	0.41	0.81	1.00	5.47	2.62	1.79	2.30
MAGs											
1	241.2	MAG-8:0	nd	nd	nd	nd	nd	0.17	0.17	0.22	0.31
2	297.2	MAG-12:0	nd	nd	nd	nd	nd	0.33	0.35	0.64	0.31
3	325.2	MAG-14:0	nd	nd	nd	nd	nd	0.38	0.42	0.63	0.48
4	351.2	MAG-16:1	nd	nd	nd	nd	nd	0.13	0.17	0.21	0.16
5	353.2	MAG-16:0	nd	nd	nd	nd	nd	1.03	1.00	1.41	2.10
6	375.2	MAG-18:3	nd	nd	nd	nd	nd	0.40	0.50	0.77	0.41
7	377.2	MAG-18:2	nd	nd	nd	nd	nd	1.16	1.34	1.45	1.24
8	379.2	MAG-18:1	nd	nd	nd	nd	nd	2.34	3.26	4.83	4.61
9	381.2	MAG-18:0	nd	nd	nd	nd	nd	0.80	0.93	1.73	1.78
10	401.2	MAG-20:4	nd	nd	nd	nd	nd	0.05	0.12	0.16	0.29
11	425.2	MAG-22:6	nd	nd	nd	nd	nd	0.33	0.55	0.81	0.55
12	427.2	MAG-22:5	nd	nd	nd	nd	nd	0.40	0.45	0.44	0.71
FFAs											
1	199.2	12:0	nd	nd	nd	nd	nd	1.18	1.62	1.96	1.99
2	227.2	14:0	nd	nd	nd	nd	nd	1.54	2.14	2.54	2.69
3	255.2	16:0	nd	1.19	1.61	2.07	2.32	10.33	13.58	14.03	15.01
4	277.2	18:3	nd	nd	nd	nd	nd	0.73	1.21	1.47	1.52
5	279.2	18:2	nd	nd	nd	nd	nd	9.51	12.25	12.92	13.16
6	281.2	18:1	nd	0.08	0.39	0.42	0.56	12.54	16.59	18.03	20.80
7	283.2	18:0	nd	2.07	2.25	2.63	3.18	7.04	9.08	11.08	11.90
8	303.2	20:4	nd	nd	nd	nd	nd	0.20	0.26	0.30	0.31
9	327.2	22:6	nd	nd	nd	nd	nd	0.36	0.70	0.84	0.85

Table S7. Concentration of phospholipid lipolysis products molecular species (μmol/g fat) in S1, L1, and L2 during in vitro digestion.

No.	m/z	Molecular species	S1			L1			L2		
			G0	G120	I120	G0	G120	I120	G0	G120	I120
PC											
1	650.5	PC (14:0/12:0)	0.02	0.01	nd	0.02	0.02	nd	0.02	0.01	nd
2	678.5	PC (14:0/14:0)	0.12	0.10	0.02	0.11	0.11	0.04	0.12	0.09	0.03
3	692.5	PC (15:0/14:0)	0.04	0.03	nd	0.03	0.03	0.01	0.03	0.03	nd
4	704.5	PC (16:0/14:1)	0.03	0.03	nd	0.03	0.03	nd	0.03	0.03	nd
5	706.5	PC (16:0/14:0)	0.88	0.73	0.19	0.79	0.77	0.32	0.84	0.72	0.27
6	720.6	PC (16:0/15:0)	0.17	0.14	0.04	0.16	0.15	0.06	0.16	0.14	0.07
7	730.5	PC (16:1/16:1)	0.06	0.05	0.01	0.06	0.06	0.05	0.06	0.06	0.05
8	732.6	PC (16:1/16:0)	0.34	0.31	0.03	0.34	0.33	0.09	0.35	0.31	0.06
9	734.5	PC (16:0/16:0)	1.07	0.94	0.46	0.97	0.93	0.52	1.01	0.90	0.70
10	744.6	PC (17:2/16:0)	0.03	0.03	nd	0.03	0.02	nd	0.03	0.03	nd
11	746.6	PC (17:1/16:0)	0.10	0.10	0.01	0.10	0.10	0.02	0.11	0.10	0.01
12	748.6	PC (17:0/16:0)	0.11	0.09	0.03	0.10	0.09	0.03	0.11	0.09	0.05
13	758.6	PC (18:2/16:0)	0.58	0.62	0.02	0.57	0.58	0.07	0.58	0.55	0.03
14	760.6	PC (18:1/16:0)	1.59	1.66	0.33	1.49	1.47	0.49	1.57	1.51	0.60
15	770.6	PC (18:3/17:0)	0.02	0.02	nd	0.02	0.02	nd	0.02	0.02	nd
16	772.6	PC (18:2/17:0)	0.04	0.04	nd	0.04	0.04	nd	0.04	0.04	nd
17	774.6	PC (19:1/16:0)	0.09	0.09	0.01	0.09	0.08	0.02	0.09	0.08	0.01
18	780.6	PC (20:5/16:0)	0.10	0.11	nd	0.09	0.09	nd	0.08	0.07	nd
19	784.6	PC (20:3/16:0)	0.36	0.37	0.03	0.38	0.37	0.06	0.40	0.37	0.05
20	786.6	PC (18:1/18:1)	1.15	1.20	0.10	1.14	1.09	0.19	1.19	1.11	0.17
21	790.7	PC (21:0/O-16:0)	0.03	0.01	0.01	0.02	0.01	0.01	0.01	0.01	0.03
22	806.6	PC (22:6/16:0)	0.05	0.05	nd	0.04	0.04	nd	0.05	0.04	nd
23	810.6	PC (20:1/18:3)	0.10	0.11	nd	0.09	0.09	0.01	0.09	0.09	nd

PI											
1	807.5	PI (18:1/14:0)	0.02	0.01	0.01	0.02	nd	nd	0.01	0.01	nd
2	833.5	PI (18:1/16:1)	0.10	0.08	0.01	0.09	0.04	nd	0.12	0.10	0.02
3	835.5	PI (18:1/16:0)	0.16	0.12	0.15	0.17	0.07	0.05	0.17	0.14	0.07
4	859.5	PI (18:3/18:0)	0.08	0.06	0.01	0.08	0.04	nd	0.08	0.06	0.01
5	861.6	PI (19:0/17:2)	0.38	0.33	0.32	0.39	0.19	0.18	0.43	0.35	0.21
6	863.6	PI (18:1/18:0)	0.53	0.46	0.48	0.54	0.25	0.27	0.60	0.49	0.30
7	883.5	PI (20:4/18:1)	0.05	0.04	0.01	0.06	0.02	nd	0.06	0.04	nd
8	885.6	PI (20:3/18:1)	0.12	0.10	0.08	0.12	0.06	0.02	0.13	0.12	0.05
9	887.6	PI (20:0/18:3)	0.08	0.07	0.08	0.09	0.04	0.03	0.09	0.08	0.02
LPC											
1	468.3	PC (14:0/0:0)	nd	0.01	0.30	nd	0.01	0.28	nd	nd	0.29
2	480.3	PC (P-16:0/0:0)	nd	nd	0.02	nd	nd	nd	nd	nd	0.01
3	482.4	PC (O-16:0/0:0)	nd	nd	0.12	nd	nd	0.10	nd	nd	0.11
4	510.4	PC (17:0/0:0)	nd	nd	0.10	nd	nd	0.09	nd	nd	0.10
5	518.3	PC (18:3/0:0)	nd	0.02	0.53	nd	0.01	0.46	nd	nd	0.43
6	520.3	PC (18:2/0:0)	nd	0.19	0.99	nd	0.12	0.74	nd	0.16	0.81
7	522.4	PC (18:1/0:0)	nd	0.34	2.55	nd	0.21	2.36	nd	0.42	2.44
8	524.4	PC (18:0/0:0)	nd	0.21	1.77	nd	0.09	1.56	nd	0.36	1.52
9	544.3	PC (20:4/0:0)	nd	nd	0.26	nd	0.01	0.23	nd	nd	0.23
PS											
1	818.6	PS (20:0/18:0)	2.29	2.18	nd	2.40	2.02	nd	2.46	2.18	0.17
2	860.6	PS (22:0/19:0)	0.10	0.09	nd	0.08	0.03	nd	0.13	0.10	nd
SM											
1	675.5	SM (d18:1/14:0)	0.22	0.20	0.21	0.23	0.20	0.23	0.27	0.24	0.23
2	677.6	SM (d18:0/14:0)	0.08	0.06	0.05	0.07	0.05	0.05	0.06	0.05	0.07
3	689.6	SM (d18:1/15:0)	0.11	0.09	0.09	0.12	0.10	0.10	0.12	0.11	0.12
4	701.6	SM (d18:1/16:1)	0.02	0.01	0.01	0.02	0.01	0.01	0.02	0.01	0.01

5	703.6	SM (d18:1/16:0)	0.99	0.95	0.98	1.02	0.89	1.13	1.08	1.02	1.15
6	705.6	SM (d18:0/16:0)	0.14	0.09	0.10	0.12	0.10	0.09	0.11	0.09	0.11
7	717.6	SM (d18:1/17:0)	0.04	0.03	0.03	0.04	0.03	0.03	0.04	0.04	0.03
8	731.6	SM (d18:1/18:0)	0.11	0.08	0.09	0.10	0.09	0.09	0.11	0.10	0.10
9	745.6	SM (d18:1/19:0)	0.03	0.01	0.01	0.02	0.02	nd	0.02	0.02	0.01
10	759.6	SM (d18:1/20:0)	0.54	0.51	0.52	0.54	0.49	0.51	0.61	0.54	0.48
11	761.7	SM (d18:0/20:0)	0.12	0.09	0.08	0.10	0.08	0.10	0.10	0.08	0.12
12	771.6	SM (d18:2/21:0)	0.04	0.03	0.03	0.04	0.03	0.02	0.04	0.03	0.03
13	773.7	SM (d18:1/21:0)	0.71	0.68	0.71	0.71	0.64	0.70	0.81	0.71	0.68
14	785.7	SM (d18:1/22:1)	0.13	0.13	0.13	0.14	0.12	0.12	0.15	0.13	0.13
15	787.7	SM (d18:1/22:0)	1.23	1.19	1.21	1.20	1.10	1.19	1.34	1.21	1.15
16	799.7	SM (d18:2/23:0)	0.10	0.10	0.11	0.10	0.09	0.11	0.12	0.10	0.11
17	801.7	SM (d18:1/23:0)	0.82	0.78	0.81	0.79	0.72	0.80	0.88	0.78	0.78
18	813.7	SM (d18:2/24:0)	0.13	0.12	0.14	0.13	0.12	0.14	0.14	0.13	0.14
19	815.7	SM (d18:1/24:0)	0.48	0.45	0.49	0.45	0.41	0.49	0.49	0.44	0.47
20	829.7	SM (d18:1/25:0)	0.05	0.04	0.05	0.05	0.04	0.04	0.05	0.04	0.04
PE											
1	688.5	PE (18:1/14:0)	0.10	0.08	nd	0.10	0.10	nd	0.10	0.09	nd
2	712.5	PE (P-18:0/17:2)	0.07	0.07	nd	0.07	0.06	0.05	0.02	0.05	nd
3	714.5	PE (18:2/16:0)	0.51	0.47	0.26	0.51	0.52	0.96	0.48	0.46	nd
4	716.5	PE (18:1/16:0)	1.12	0.92	0.46	0.98	0.97	0.10	1.20	0.85	nd
5	738.5	PE (22:4/14:0)	0.29	0.27	nd	0.28	0.26	nd	0.19	0.24	nd
6	740.5	PE (20:3/16:0)	0.92	0.86	nd	1.01	1.00	0.05	0.84	0.89	nd
7	742.5	PE (18:2/18:0)	3.96	3.54	0.70	3.82	3.73	0.47	3.86	3.28	2.52
8	766.5	PE (20:3/18:1)	0.16	0.15	nd	0.16	0.16	nd	0.12	0.14	nd
9	790.5	PE (22:6/18:0)	0.01	0.02	nd	0.02	0.01	nd	nd	0.01	nd
LPE											
1	436.3	PE (P-16:0/0:0)	nd	nd	0.07	nd	nd	0.03	nd	nd	0.05

2	452.3	PE (16:0/0:0)	nd	0.11	1.01	nd	0.03	0.71	nd	0.07	0.77
3	464.3	PE (P-18:0/0:0)	nd	nd	nd	nd	nd	nd	nd	nd	0.01
4	476.3	PE (18:2/0:0)	nd	0.06	0.26	nd	0.10	0.16	nd	0.06	0.20
5	478.3	PE (18:1/0:0)	nd	0.37	2.84	nd	0.25	2.08	nd	0.34	2.15
6	480.3	PE (18:0/0:0)	nd	0.19	1.57	nd	0.06	1.07	nd	0.13	1.14
7	500.3	PE (20:4/0:0)	nd	0.01	0.17	nd	0.02	0.09	nd	nd	0.14
Others			nd	nd	1.32	nd	nd	1.00	nd	nd	1.67

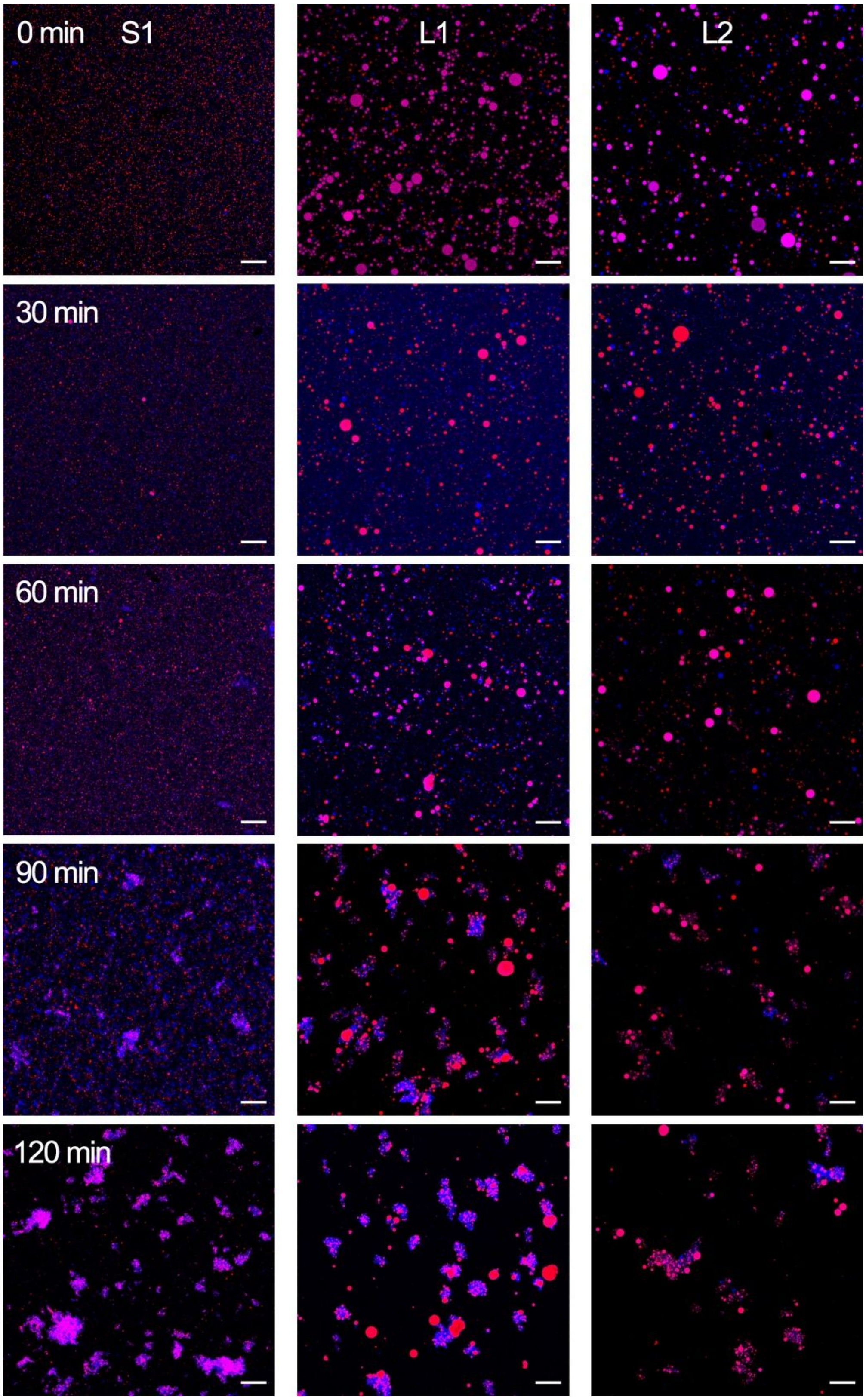

Figure S1. CLSM images of S1, L1, and L2 at 0, 30, 60, 90, and 120 min during in vitro gastric digestion. Neutral lipids strained with Nile Red (red), and glycoproteins & glycolipids stained with WGA (blue). All scale bar, 20 μm.

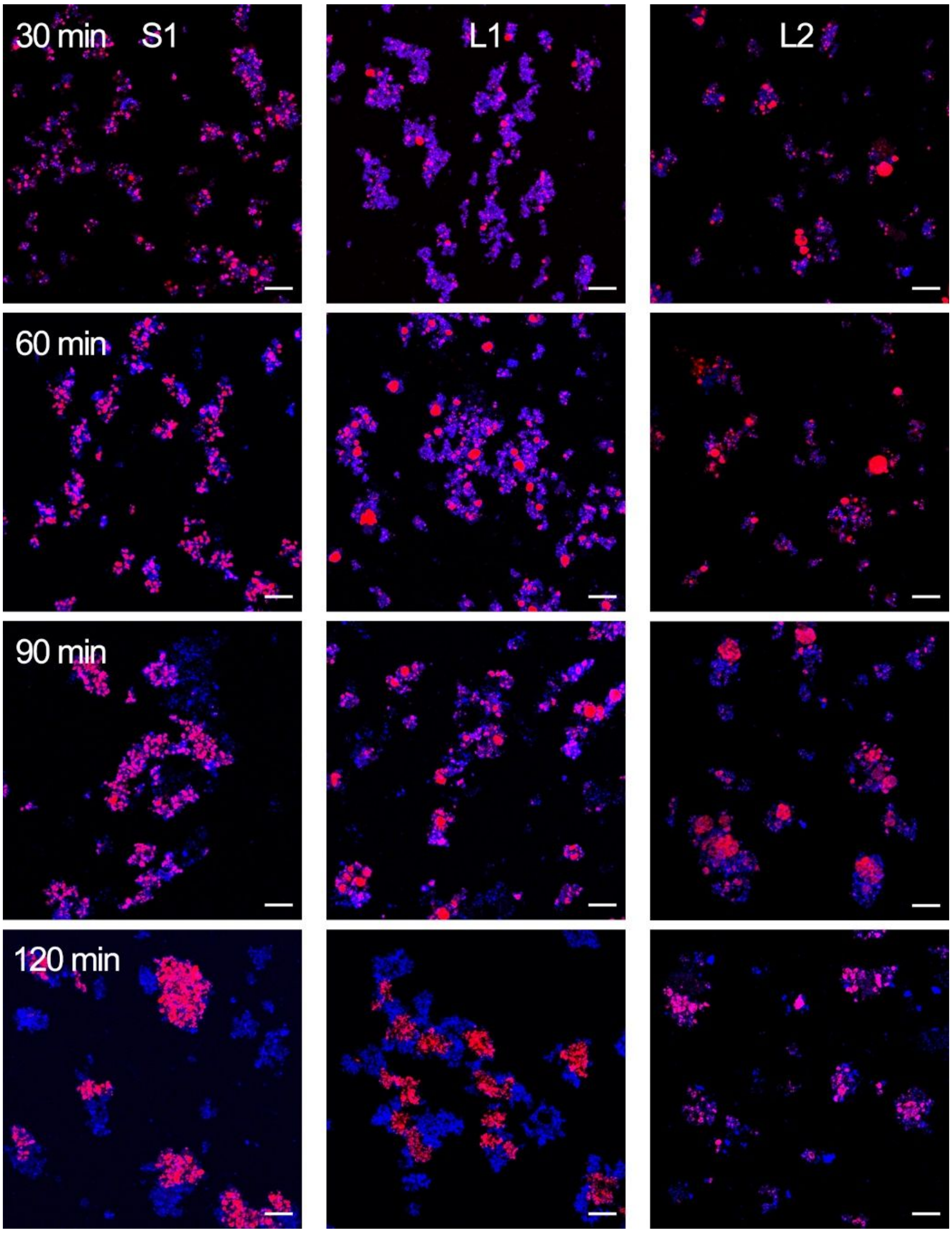

Figure S2. CLSM images of S1, L1, and L2 at 0, 30, 60, 90, and 120 min during in vitro intestinal digestion. Neutral lipids strained with Nile Red (red), and glycoproteins & glycolipids stained with WGA (blue). All scale bar, 20 μm.
